# A streamlined tandem tip-based workflow for sensitive nanoscale phosphoproteomics

**DOI:** 10.1038/s42003-022-04400-x

**Published:** 2023-01-18

**Authors:** Chia-Feng Tsai, Yi-Ting Wang, Chuan-Chih Hsu, Reta Birhanu Kitata, Rosalie K. Chu, Marija Velickovic, Rui Zhao, Sarah M. Williams, William B. Chrisler, Marda L. Jorgensen, Ronald J. Moore, Ying Zhu, Karin D. Rodland, Richard D. Smith, Clive H. Wasserfall, Tujin Shi, Tao Liu

**Affiliations:** 1 Biological Sciences Division, Pacific Northwest National Laboratory, Richland, WA 99354 USA; 2 Institute of Plant and Microbial Biology, Academia Sinica, Taipei, Taiwan; 3 Environmental Molecular Sciences Laboratory, Pacific Northwest National Laboratory, Richland, WA 99354 USA; 4 Department of Pathology, Immunology, and Laboratory Medicine, Diabetes Institute, College of Medicine, University of Florida, Gainesville, FL 32611 USA

**Keywords:** Proteomics, Mass spectrometry, Proteomics

## Abstract

Effective phosphoproteome of nanoscale sample analysis remains a daunting task, primarily due to significant sample loss associated with non-specific surface adsorption during enrichment of low stoichiometric phosphopeptide. We develop a tandem tip phosphoproteomics sample preparation method that is capable of sample cleanup and enrichment without additional sample transfer, and its integration with our recently developed SOP (Surfactant-assisted One-Pot sample preparation) and iBASIL (improved Boosting to Amplify Signal with Isobaric Labeling) approaches provides a streamlined workflow enabling sensitive, high-throughput nanoscale phosphoproteome measurements. This approach significantly reduces both sample loss and processing time, allowing the identification of >3000 (>9500) phosphopeptides from 1 (10) µg of cell lysate using the label-free method without a spectral library. It also enables precise quantification of ~600 phosphopeptides from 100 sorted cells (single-cell level input for the enriched phosphopeptides) and ~700 phosphopeptides from human spleen tissue voxels with a spatial resolution of 200 µm (equivalent to ~100 cells) in a high-throughput manner. The new workflow opens avenues for phosphoproteome profiling of mass-limited samples at the low nanogram level.

## Abstract (web summary)

A streamlined tandem tip-based workflow for sensitive nanoscale phosphoproteomics is developed, reducing sample loss and processing time, allowing the phosphoproteome profiling of mass-limited samples at the low nanogram level.

## Introduction

The ability to trace dynamic protein phosphorylation in small populations of cells can reveal cell-to-cell heterogeneity in cell signaling (e.g., cellular responses to stimulus), and provide a foundation for identifying rare cell types within a clinical sample and better understanding of disease mechanisms. Conventional phosphoproteomic approaches require large sample sizes, which obscures cell type-specific signaling information. Sensitive and nanoscale phosphoproteomic methods have the potential to make such cell type-specific applications more feasible in clinical settings, and also enable more precise phenotypic analysis of cell (sub)types in a time- and/or dose-dependent fashion. Indeed, mass spectrometry (MS)-based single-cell proteomics has recently made significant advancements in detection sensitivity and high-throughput measurements^1–4^. A remaining technical challenge for nanoscale proteomics is the comprehensive quantitative profiling of post-translational modifications (PTMs), particularly protein phosphorylation states^5^, key indicators for signaling pathways and network activations essential for many physiological functions^6,7^.

The combination of metal affinity chromatography (e.g., Fe(III) and TiO_2_) with extensive sample fractionation has made it possible to identify and quantify >30,000 phosphorylation sites from bulk materials (over mg of proteins)^8–10^. To extend phosphoproteomics measurement to small sample, Humphrey et al.^11^ described an EasyPhos protocol to facilitate rapid and reproducible phosphopeptide enrichment. They also modified the buffer during digestion and employed extended MS/MS accumulation times to enhance phosphopeptide detection in small-sized samples. Starting with 12.5 μg and 25 μg of peptides, approximately 8000 and 9000 phosphopeptides could be identified, respectively^11,12^. Similarly, Post et al.^13^ assessed an automated enrichment protocol using Fe(III)-IMAC (immobilized metal affinity chromatography) cartridges on a AssayMAP Bravo platform to identify approximately 1500 and 4500 phosphopeptides from 1 μg and 10 μg of HeLa cell digests, respectively^13^. Chen et al.^14^ developed an integrated strategy, termed Phospho-SISPROT to identify approximately 600 and 3000 phosphopeptides from 1 μg and 10 μg of HEK293T cell digests, respectively. However, technical challenge remains for effective phosphopeptide enrichment and deep phosphoproteome profiling from low µg and sub-μg sample sizes.

Recently, a signal boosting strategy utilizing multiplexed isobaric labeling, in which a large amount of relevant boosting (or carrier) material is labeled with one or several of the isobaric (e.g., tandem mass tag; TMT) reagent(s) and then mixed with the labeled low-amount samples using other TMT channels, has enabled single-cell proteomics analysis with enhanced detection sensitivity and sample throughput^15–17^. This strategy has been applied to enhance the detection of phosphopeptides in low-amount samples. It enabled the reliable quantification of >20,000 phosphorylation sites in 150 human pancreatic islets^18^ and >2300 low-abundance phosphotyrosine peptides from 1 mg of T cell receptor stimulated Jurkat T cells^19^.

To enable the sensitive phosphoproteome analysis of nanogram samples, herein we developed a streamlined tandem tip-based sample preparation workflow, which integrates surfactant-assisted one-pot (SOP) digestion, tandem tip-based C18-IMAC-C18 enrichment/cleanup, and improved boosting to amplify signal with isobaric labeling (iBASIL) for MS data acquisition. The tandem tip method significantly reduces sample loss and increases throughput. Its analytical merits were benchmarked using 0.1, 1 and 10 ng peptides samples (equivalent to single cells, 10 cells, and 100 cells, respectively) from 3 different acute myeloid leukemia (AML) cell lines. The performance of the workflow was further demonstrated in the analysis of 100 fluorescence-activated cell sorting (FACS)-sorted MCF10A cells before and after EGF treatment and laser-dissected human spleen tissue voxels (equivalent to ~100 cells). This workflow can recapitulate the dynamic changes in protein phosphorylation in the small-sized samples, demonstrating its potential for broader applications in biological and biomedical research.

## Results

### The integrated tandem tip-based nanoscale phosphoproteomics workflow

**Fig. 1:**
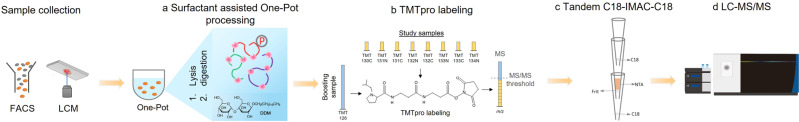
A streamlined tandem tip-based workflow for nanoscale phosphoproteomics. **a** The proteins are digested by our recently developed SOP (Surfactant-assisted One-Pot) approach. **b** The digested peptides are labeled with TMTpro reagents for sample multiplexing. **c** TMT labeled phosphopeptides are purified by tandem tip-based C18-IMAC-C18. **d** The enriched phosphopeptides are analyzed by LC-MS/MS applying iBASIL settings.

Similar to single-cell proteomics^20^, the major challenge for nanoscale phosphoproteomics is the substantial sample loss due to non-specific surface adsorption during multi-step sample transfer steps. To address this issue, we have developed a streamlined tandem tip-based workflow for sensitive nanoscale phosphoproteomics (Fig. 1) which effectively integrates four components: (1) a sample lysis and digestion by SOP processing^20^, (2) isobaric labeling/boosting using TMTpro (16-plex), (3) phosphopeptide enrichment/cleanup using a tandem C18-IMAC-C18 tip arrangement, and (4) improved MS data acquisition using the iBASIL strategy^17^. The streamlined nanoscale phosphoproteomic workflow primarily benefits from the new tandem tip C18-IMAC-C18 method that maximizes phosphopeptide recovery from IMAC enrichment of low-input samples.

Conventional workflows for phosphopeptide enrichment have generally been developed for bulk sample analysis with typical ≥100 μg sample inputs^10^. They are usually performed in a sample vial (i.e., in-vial processing)^10^, involve multiple sample transfers, use large processing volumes, and incur long processing times. Our alternative tandem tip approach integrated C18 tip-based peptide desalting with IMAC-based phosphopeptide enrichment, followed by direct LC-MS analysis without sample transfer steps. The Ni-NTA silica beads were packed into the pipette tip to form tip IMAC. The C18 elution buffer for the desalting procedure (80% ACN in 0.1% TFA) is also used as the IMAC loading buffer. The IMAC elution buffer (NH_4_H_2_PO_2_ at pH 4.4) is compatible with reversed-phase C18 for sample loading, which eliminates the step of peptide lyophilizing. The buffer compatibility makes the tandem C18-IMAC-C18 method ideal for phosphopeptide enrichment from small samples and subsequent LC-MS/MS analysis.

### Tip-IMAC vs. in-vial IMAC

**Fig. 2:**
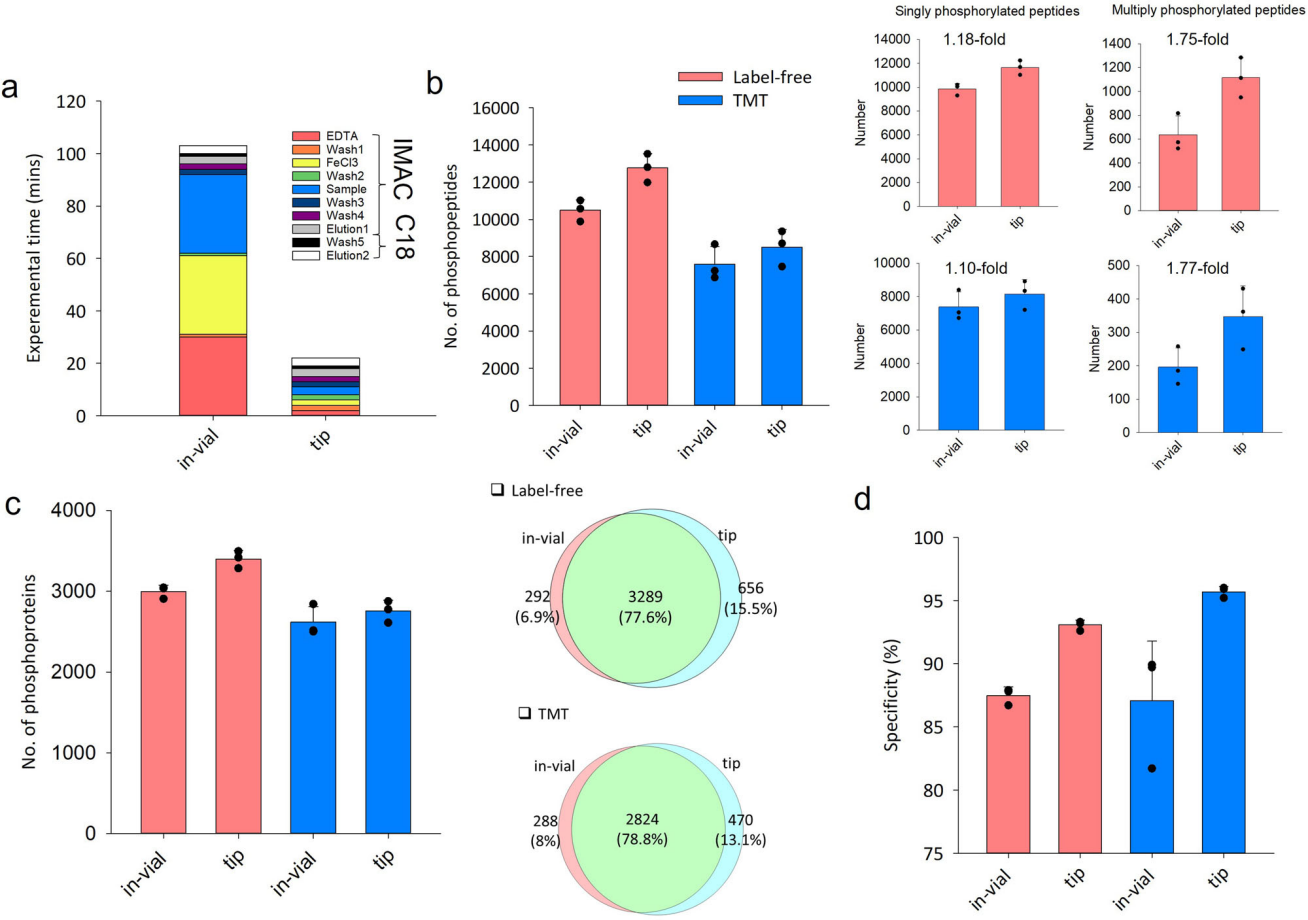
Comparison of in-vial and tip-based IMAC for phosphopeptide enrichment. **a** The processing time for in-vial and tip-based IMAC. The numbers of identified phosphopeptides (**b**), phosphoproteins (**c**) and the specificity of phosphopeptide enrichment (**d**) for unlabeled and TMT-labeled peptides from MCF-7 cells using the in-vial and tip-based IMAC are shown. Sample amount: 150 μg of peptides. The error bars represent the standard deviation (*n* = 3).

Compared to in-vial phosphopeptide enrichment, tip-IMAC reduced the total processing time from ≥100 min to ~20 min (Fig. 2a) and thus increased the analytical throughput by 5X. The performance of tip-IMAC step was evaluated using both unlabeled and TMT-labeled peptides. The same amount of MCF-7 peptides was aliquoted for tip-IMAC and in-vial IMAC^10^. When comparing the tip-IMAC protocol with in-vial IMAC protocol^10^ (Fig. 2b), we observed 22% (from 10,490 to 12,769) and 12% (from 7590 to 8504) increases in the numbers of phosphopeptide identification, and 13% and 5% increases in the numbers of identified phosphoproteins (overlap was greater than 77%) (Fig. 2c), for samples without and with TMTzero labeling, respectively. Besides the increased number of phosphopeptides, the tip-IMAC method resulted in significantly increased coverage of multiply phosphorylated peptides in both label-free and TMT labeling analyses (Fig. 2b) and higher specificity (>90%) of phosphopeptide purification (Fig. 2d).

### Tandem tip vs. step-by-step enrichment

We implemented an integrated tandem tip-based method (C18-IMAC-C18) to avoid sample transfer between different vials and further increase sample recovery (Fig. S1a). The digested peptides from 10 μg of A549 cell lysates were purified in parallel by the tandem tip and conventional (step-by-step) methods. Compared to the step-by-step method, the tandem tip method improved by 7% in the number of identified phosphopeptides (from 9115 to 9720, *p* = 0.01), by 3% in the number of identified phosphoproteins (from 2827 to 2880, *p* = 0.002), and by 3% in the XIC area (Fig. S1b). Moreover, this tandem tip method also provided improved Pearson correlation with lower quantitation CV (coefficient of variation) (Fig. S1c). To demonstrate that it is also suitable for robust analysis at higher input levels, we evaluated the inter-day and intra-day reproducibility of this integrated C18-IMAC-C18 method using more precise selected reaction monitoring (SRM)-based targeted proteomics approach. We spiked 10 SIL phosphopeptide mixture at 2 µM concentration into reference endometrial tumor digest (200 µg) for tandem tip purification. Its high reproducibility across different batches and days was evident by the Pearson correlation (*r* > 0.99) and CV (median = 15.8%) (Fig. S2).

### Tandem IMAC-HpH-C18 for phosphopeptide fractionation

**Fig. 3:**
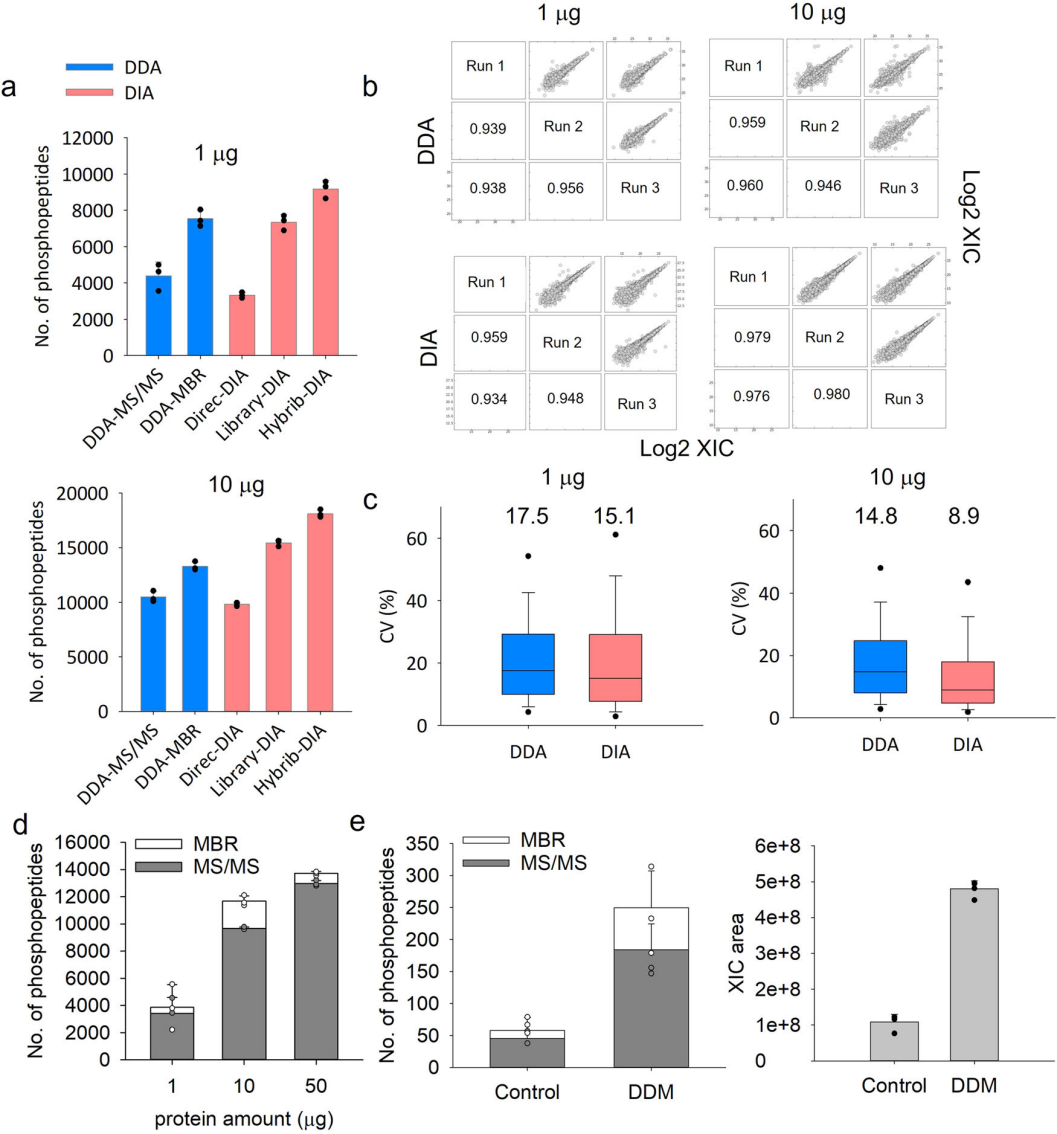
Evaluation of the performance of tandem C18-IMAC-C18. The number of phosphopeptides (**a**), reproducibility (Pearson correlation) (**b**) and CV (%) (**c**) of phosphoproteomics analyses of 1 and 10 μg of proteins from mixture of 3 AML cells using single-shot DDA and DIA. A phosphoproteome spectrum library constructed from data described in Fig. S3c, d was used for Library DIA and MBR DDA analysis. **d** The numbers of identified phosphopeptides (with and without MBR) from 1, 10 and 50 μg of A549 cell lysates. **e** The numbers of identified phosphopeptides (with and without MBR) and XIC area for 0.1 μg of A549 cell lysates with and without DDM. The error bars represent the standard deviation (*n* = 3).

The home-made tip IMAC can be directly integrated with a high-pH C18 reversed-phase (HpH-C18) tip^21^ for in-tip phosphopeptide fractionation. Purified phosphopeptides from 20 μg peptides were directly fractionated by HpH-RP tip (Fig. S3a) into 4 fractions, and the high separation efficiency (only 0.9% overlapping phosphopeptides across 4 fractions) by HpH-RP tip (Fig. S3b) effectively reduced sample complexity. The integrated IMAC-HpH-RP tip can be also used for large sample size (e.g., ≥100 μg) phosphopeptide fractionation. The phosphopeptides from 500 μg peptides were firstly purified by tip-IMAC and directly fractionated into 6 fractions by HpH-RP tip^21^ (Fig. S3c). The strong fractionation capability of this method (Fig. S3d) is evident by the low overlap of identified phosphopeptides between fractions and the overall comprehensive phosphoproteome coverage (46,703 identified phosphopeptides and 6095 phosphoproteins) from only 500 μg peptides. This is especially useful for generating spectral libraries for highly effective data-dependent acquisition (DDA) and data-independent acquisition (DIA) analysis (see Fig. 3 below). These results demonstrated that tandem C18-IMAC-C18 can be broadly used not only for sensitive phosphoproteomic analysis of small samples but also as a convenient fractionation system (without the need of LC) to achieve in-depth phosphoproteome coverage.

### Evaluation of tandem C18-IMAC-C18 detection sensitivity using label-free methods

We first evaluated the detection sensitivity of tandem tip using two different label-free methods (DDA and DIA). The tip-IMAC allowed for identification of 4394/3330 and 10,482/9831 phosphopeptides in 1 and 10 μg of tryptic peptides by single-shot DDA/direct DIA without library search or match-between-run (MBR), respectively (Fig. 3a). MBR is a common approach to mitigate the missing value problem, where peptides identified by tandem mass spectra in one run are transferred to another by inference based on m/z, charge state, and retention time. To improve the phosphoproteome coverage, we used the phosphopeptides identified from the above HpH-C18 tip-fractionated 500-µg sample (Fig. S3c, d) to construct a spectral library. This enabled a substantial increase of phosphopeptide identifications: 7535/9180 and 13,268/18,100 phosphopeptides in 1 and 10 μg peptide samples by DDA (MBR)/DIA (library search), respectively (Fig. 3a); DIA also was demonstrated with better quantitation quality as indicated by low protein CVs (Fig. 3b, c). These results suggest that the tandem C18-IMAC-C18 method is highly reproducible for low-input phosphoproteomics analysis (e.g., 1 and 10 μg).

We next evaluated the detection sensitivity of the tandem C18-IMAC-C18 method using DDA analysis of A549 with 1-, 10-, and 50-μg protein input (Fig. 3d), as well as an even lower input of 0.1-μg (equivalent to 1000 cells). The tandem tip method allowed for identification of 12,975 and 9675 phosphopeptides from 50 and 10 µg of starting material, respectively, and 3412 phosphopeptides from 1 µg of starting material. After using the MBR function across different samples, the number of identified phosphopeptides was increased to 4477, 12,824 and 15,754 from 1, 10, and 50 μg, respectively.

We also assessed the effect of DDM by single-shot DDA analysis (Fig. 3e), which has been previously demonstrated to greatly reduce surface adsorption loss in single-cell and low-input proteomics^20,22,23^, for enhancing phosphopeptide detection at sub-μg input level using the tandem tip method. 0.1 μg of cell lysates were digested in the PCR tubes without and with DDM treatment (i.e., the SOP approach^20^), and loaded into tandem tip (C18-IMAC-C18) for phosphopeptide enrichment with and without DDM treatment. With the surface protection by DDM, the number of identified phosphopeptides was substantially increased from 45 to 184 based on the MS/MS (or 58 to 250 based on MBR), with ~4.4-fold enhancement in MS signals (XIC areas) (Fig. 3e). In summary, we demonstrated that the tandem tip method enables robust and comprehensive phosphoproteome coverage for ≥1 μg of proteins. However, the coverage is still limited for sub-µg input levels even when spectrum library is used for matching, presumably due to the lower MS1 signals.

### Integration of tandem C18-IMAC-C18 method with iBASIL

**Fig. 4:**
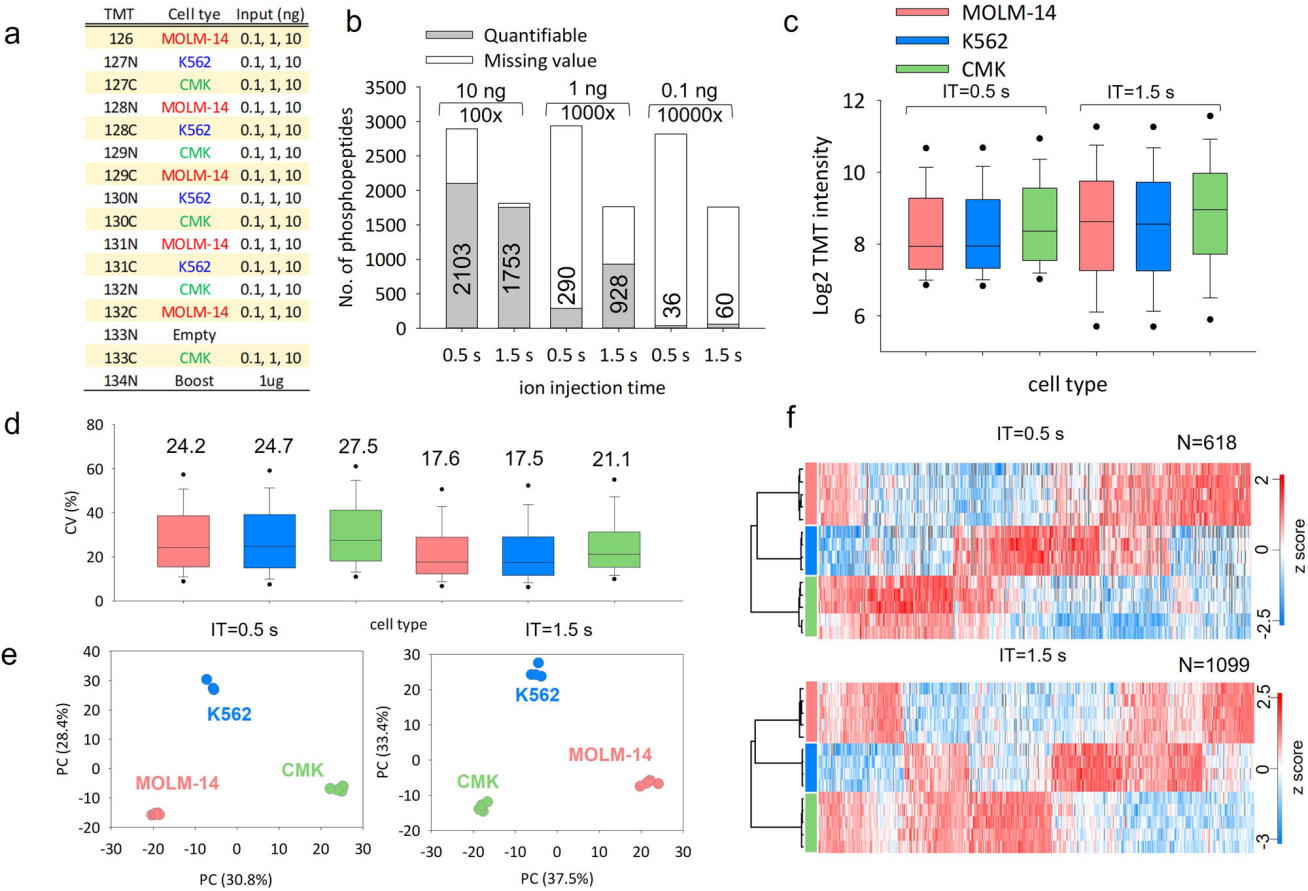
Mimic nanoscale phosphoproteome analysis using tip-IMAC and iBASIL. **a** The TMT experiment design for the phosphoproteomics analysis of tryptic digests (0.1, 1 and 10 ng) from three different types of AML cells (MOLM-14, K562 and CMK). **b** The numbers of quantified phosphopeptides (70% no-missing value in study samples) from 0.1, 1 and 10 ng of tryptic digests under different ion injection times (0.5 and 1.5 s). The TMT reporter ion intensity distribution (**c**), CV (%) of replicate experiments (**d**), PCA analysis (**e**), and heatmap of significantly changed phosphopeptides of the 10-ng input samples under different ion injection times (**f**).

To further increase the detection sensitivity for sub-μg input levels, we integrated the tandem C18-IMAC-C18 method with our recently developed carrier proteome-based iBASIL approach^17,18^ (Fig. 1d). To mimic nanoscale phosphoproteome analysis, 0.1, 1 and 10 ng of tryptic digest of 3 different AML cells (MOLM-14, K562 and CMK) equivalent to total protein content in 1, 10 and 100 cells, respectively, were analyzed in 3 separate TMT-16 experiments, each with 1 µg of the same tryptic digest of mixed cells as the boosting sample (Fig. 4a). The 3 sets of TMT16-labeled samples were then processed by tandem tip. To minimize the ion under sampling effect caused by the overwhelming amount of ions from the boosting channel^17,24^, high AGC (1E6) and long ion injection time (IT, 0.5 s and 1.5 s) were used to improve the sampling of phosphopeptide ions in sample channels. Approximately 2800 phosphopeptides were identified for all three TMT-16 experiments, but only 36, 290 and 2103 phosphopeptides were quantified (70% valid values in one cell type) from the 1-, 10- and 100-cell analysis using the 500-ms IT time setting, respectively (Fig. 4b). Increasing the IT time from 0.5 s to 1.5 s increased the cycle time, resulting in decreasing the number of identified phosphopeptides down to ~1700; however, higher IT time effectively improved the ion sampling from the samples in the sample channel, resulting in significantly higher number of quantified phosphopeptides (e.g., from 290 to 928 for the 10-cell analysis) (Fig. 4b). Higher IT time slightly decreased the number of quantifiable phosphopeptides for the 100-cell (10 ng) analysis (Fig. 4b), presumably due to the overall reduced number of PSMs as a result of the prolonged duty cycle. Wider dynamic range of TMT reporter ion intensity was obtained by increasing the IT time (Fig. 4c). The higher IT time also improved the quantitation precision in CV (%) (Fig. 4d) and separation of the three AML cell types in 100-cell analyses using the resulting phosphoproteome profiles (Fig. 4e). Moreover, the higher quality quantitative results (Fig. 4f) via higher IT time generated more statistically significant phosphopeptides amongst the three types of AML cells (618 and 1099 phosphopeptides with adjusted *p* value < 0.05 using the 0.5-s and 1.5-s methods, respectively), resulting in increased coverage in signaling pathways (Fig. S4).

Up to 928 phosphopeptides can be quantified in the 10-cell analysis (1 ng of peptides); however, the three AML cell types did not cluster tightly in the principal component analysis (PCA), especially when lower IT time was used (Fig. S5). The outlier data points were from three TMT sample channels (TMT132N, 132 C and 133 C), most likely due to isotopic impurities from the TMT134N channel where the boosting material was labeled at 1000-fold higher input level. Similar result was observed in previous study where TMT-10/11/16 was used^17,25^. This suggests that more channels adjacent to the boosting channel should be left empty when a high boosting ratio is used (e.g., ≥1000x). Nevertheless, these results demonstrated the utility of the integrated C18-IMAC-C18/iBASIL platform for robust quantitative nanoscale phosphoproteomic analysis of 10–100 cells.

### Streamlined SOP/C18-IMAC-C18/iBASIL workflow for phosphoproteomics analysis of 100 sorted cells

**Fig. 5:**
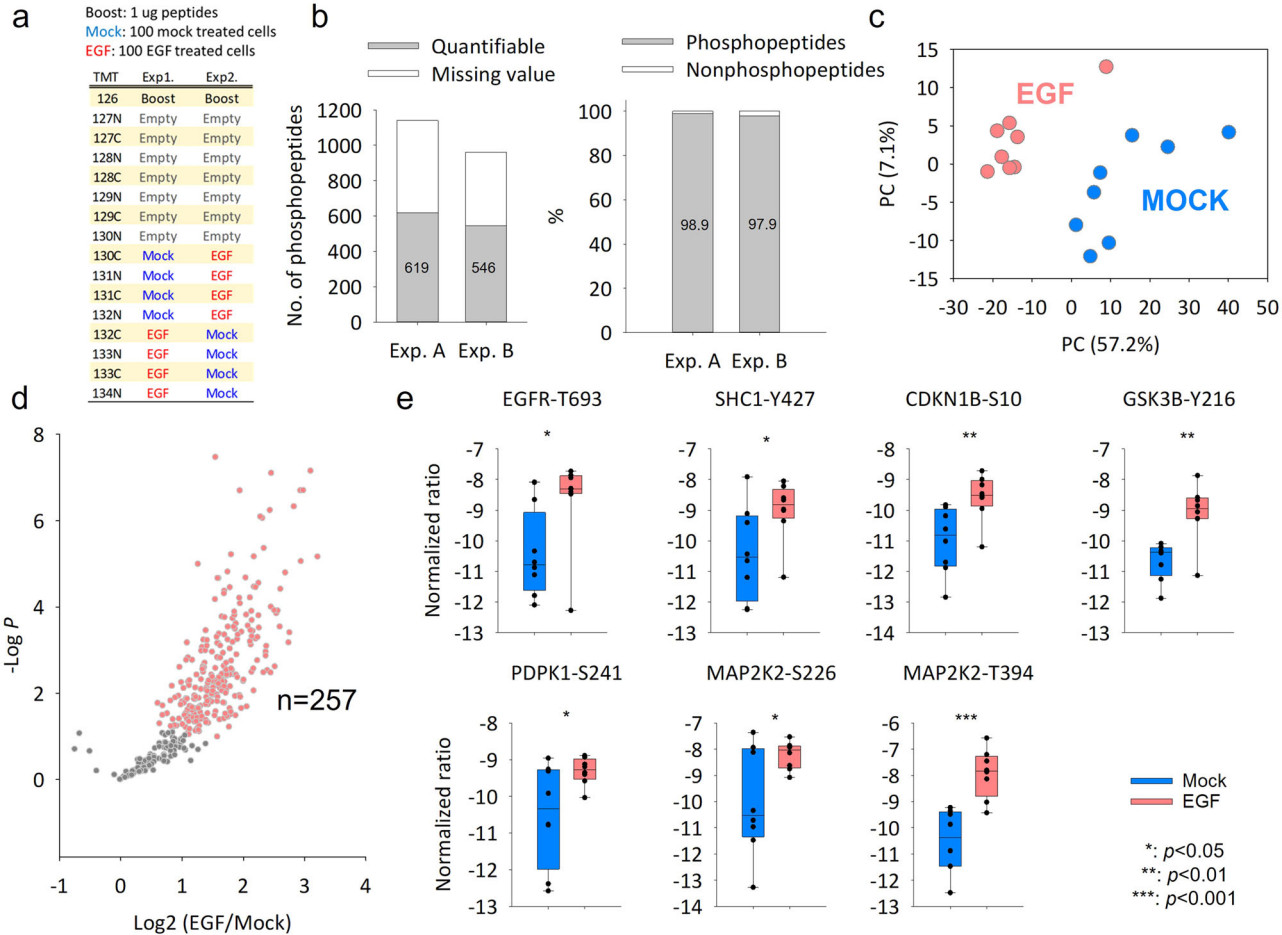
Phosphoproteome analysis of 100 MCF10A cells sorted by FACS using the streamlined SOP/C18-IMAC-C18/iBASIL workflow. **a** The TMT experiment design. **b** The number of quantified phosphopeptides (70% no-missing value in study samples) and the enrichment specificity in each TMT experiment. **c** PCA analysis shows the cells cluster under the two different conditions. **d** Volcano plot shows significantly changed phosphopeptides between EGF-treated cells and mock cells (*t*-test, *n* = 8 for each condition; s0 = 1 and FDR = 0.05% were used as cut-off values) (**e**) The significantly altered phosphorylation sites in the ErbB signaling pathway. *Y*-axis means the normalized TMT intensity after medium centering and batch correction by Combat (*t*-test, *n* = 8).

To explore the full potential of the streamlined nanoscale phosphoproteomics platform, small numbers of cells sorted by FACS were used to evaluate its performance. 100 FACS-sorted MCF10A cells with and without EGF treatment were processed by SOP method, followed by 16-plexed TMTpro labeling (130 C to 134 N). To avoid the potential isotopic leakage issue^17,24^ (e.g., in the 10-cell analysis in Fig. S5), the TMT-labeled 100-cell samples were mixed with 1 µg of pre-digested tryptic peptides from EGF-treated MCF10A cells that was labeled in a far separate channel (TMT126) while leaving the TMT channels 127 N to 130 N empty (Fig. 5a). The labeled peptides were directly loaded into tandem tip C18-IMAC-C18 for phosphopeptide enrichment/cleanup, and the resulting samples were analyzed by LC-MS/MS with the iBASIL settings (i.e., both high AGC and IT time that can increase the quantitation accuracy and detection sensitivity^24^).

Approximately 1000 phosphopeptides were identified with ~600 quantifiable sites in >70% of the study samples using half of the resulting sample with the conventional LC-MS platform (i.e., the Lumos Orbitrap MS and 75-µm i.d. LC column). High specificity (>98%) was achieved for phosphopeptide enrichment (Fig. 5b), which helped reduce potential dynamic range compression due to co-isolated non-phosphopeptides during MS/MS sampling. The quantified phosphopeptides allowed for robust separation of 100 sorted MCF10A cells (single-cell level input of the enriched phosphopeptides) with and without EGF treatment (Fig. 5c) and 257 phosphopeptides were found to be upregulated upon EGF treatment (Fig. 5d and Supplementary Data 2). The altered phosphopeptides were subjected to KEGG and Reactome pathway analysis using DAVID^26^ and STRING^27^, respectively. As expected, known pathways (e.g., ErbB and insulin signaling pathways; Supplementary Data 2) were enriched. We identified 7 key upregulated phosphorylation sites in ErbB signaling pathways, such as T693 in EGFR^28^, Y427 in SHC1^29^, S10 in CDKN1B^30^, Y216 in GSK3B^31^, S241 in PDPK1^32^, and S226^33^ and T394 in MAP2K2^34,35^ (Fig. 5e). In addition, mRNA splicing was found to be activated after EGF treatment (Supplementary Data 2). In a previous study, Zhou et al.^36^ described that EGF signaling promotes changes in splicing via AKT and SRPKs, which is consistent with our finding in this study. We also tried to analyze 10 sorted MCF10A cells with and without EGF treatment (Fig. S6). While the different cell states (i.e., with and without EGF treatment) can be separated by PCA analysis, the coverage of quantifiable phosphopeptides were limited (*n* < 200; enrichment specificity at >98% level), likely due to the insufficient detection sensitivity (Fig. S6).

### Spatial phosphoproteomics and proteomics analysis using the streamlined workflow

**Fig. 6:**
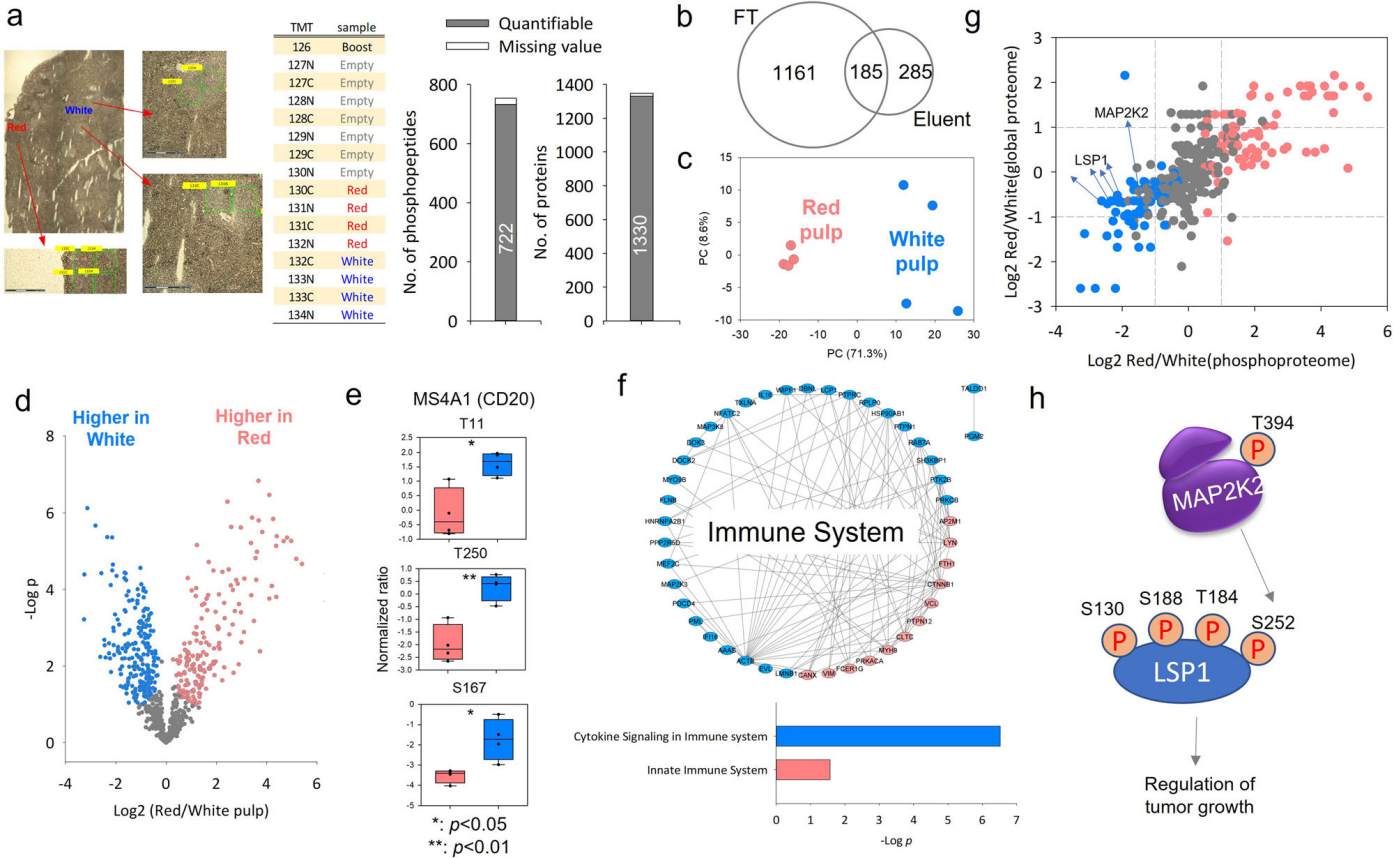
Phosphoproteome analysis of LCM-dissected human spleen tissue voxels. **a** The optical image and the TMT experiment design of the white (TMT132C, TMT131N, TMT133C, and TMT134N), and red pulp regions (TMT130C, TMT131N, TMT131C, and TMT132N), and the number of quantified phosphopeptides from IMAC eluent and proteins from IMAC flow-through (FT). **b** The overlap of quantified proteins between IMAC eluent and FT. **c** PCA of the phosphoproteome data. **d** Volcano plot (*t*-test, *n* = 8 for each condition; s0 = 1 and FDR = 0.05% were used as cut-off values) shows significantly changed phosphopeptides in white pulp and red pulp. **e** The significantly altered phosphorylation sites in the MSFA1 (CD20) proteins. **f** The annotated pathway for the significantly up-regulated phosphorylation peptides in white or red pulp regions by STRING. **g** The quantitation correlation between global and phosphoproteome. **h** Known signaling transduction for triggering apoptosis in activated B cells.

We next tested the utility of the streamlined workflow for spatial phosphoproteomics analysis using small human spleen tissue voxels (Fig. 6). Two distinct regions, red and white pulps, were dissected by laser capture microdissection (LCM) to generate small tissue voxels (Fig. 6a). Each tissue voxel has a size of 200 µm × 200 µm with 10 µm in thickness (approximately 100 cells^22^). They were first processed by convenient microPOTS (microdroplet processing in one pot for trace samples) at the low µL processing volume^37^. The streamlined C18-IMAC-C18/iBASIL workflow was then applied for nanoscale phosphoproteomics analysis.

Importantly, with TMT labeling and analysis of both the IMAC-enriched fraction and IMAC flow-through, we can characterize the phosphoproteome and proteome of each tissue voxel in an integrated fashion. A total of 737 unique phosphopeptides (470 phosphoproteins) were identified with 84% specificity from IMAC eluent, and 1346 proteins (6216 peptides) were identified from the IMAC flow-through (Fig. 6a). Compared to the global proteome from the flow-through fraction, an additional 285 less abundant proteins were identified through IMAC enrichment (Fig. 6b), such as the G protein-coupled seven transmembrane receptor CXCR5 and the transcription factor TFAP4. The red and white pulp regions could be readily separated in PCA using either the phosphoproteome data (Fig. 6c) or the proteome data (Fig. S7a). Analysis of variance (ANOVA) revealed that 56% of the quantified phosphopeptides (Fig. 6d; FDR < 0.05) and 69% of the quantified proteins (Fig. S7b; FDR < 0.05) were differentially expressed between the white and red pulp regions, including 11 CD markers (Fig. S7c).

Red pulp is a loose spongy tissue with chords of reticular cells located between venous sinuses that primarily contain erythrocytes, with lymphocytes, macrophages, granulocytes, and plasma cells potentially trafficking through. Uniquely in the red pulp of human spleen CD8a lines these sinuses, termed littoral cells^38^, and also marks a subset of T cells through the spleen (CD8a red in Fig. S7d). CD3e marks both CD8 and CD4 T cells interacting in follicles and dispersed throughout the organ (CD3e white in Fig. S7d). This is the major site for both erythrocyte storage and removal of bound immune complexes through complement receptors. In addition, senescent erythrocytes are destroyed by macrophages with hemoglobin:haptoglobin complexes being loaded onto CD163 positive macrophages for metabolic processing. This is consistent with the observed expression of surface protein markers (e.g., CD99, CD47 and CD55) as well as other protein markers (e.g., haptoglobin and CD163) at higher levels in red pulp, with the platelet-related pathway enriched (Supplementary Data 3).

In contrast, white pulp is composed of lymphoid follicles with central B lymphocytes (CD20 green in Fig. S7d), interacting CD3 T cells and follicular dendritic cells orchestrating immune responses. There is also a collection of lymphocytes surrounding small arterioles (CD31 blue in Fig. S7d), called periarteriolar lymphoid sheaths (PALS)^39^. CD20 is a B cell-specific membrane protein and represents an attractive target for therapeutic antibodies^40^. As expected, CD20 protein (Fig. S7c) and its phosphorylation site (Fig. 6e) have higher expression levels in the white pulp. Both white and red pulps contain lymphocytes, neutrophils, macrophages, and other innate lymphoid cells^39^.

Corresponding pathways relevant to these cell types were observed to be enriched in both regions of spleen tissues (e.g., immune system; Supplementary Data 3 and Fig. 6f). Figure 6f shows the protein-protein interaction of the significantly up-regulated phosphorylation peptides in white or red pulp regions and the enriched pathways using STRING. However, cytokine signaling was enriched in white pulp, reflecting the role of T cell derived cytokines in triggering maturation of B cells in white pulp, while proteins related to the innate immune system were enriched in red pulp, reflecting ongoing immune reactions in red pulp (Fig. 6f). Interestingly, both LSP1 and an upstream kinase MAP2K2 had significant increase at phosphorylation site levels but not at protein level in the white pulp (Fig. 6g). LSP1 is a key switch that triggers apoptosis in activated B cells^41^ (Fig. 6h); increased MAP2K2 phosphorylation is consistent with the increased proliferation of immature B cells in response to the cytokines released by T cells in the white pulp.

Taken together, our results demonstrate unprecedented data quality in integrated, high-resolution spatial phosphoproteomics and proteomics analysis using the streamlined nanoscale proteomics workflow within an exemplar spleen organ. This approach has the potential to be an enabling tool for spatial phosphoproteomics profiling. These results also recapitulated at the protein level the cell type distribution and function of white pulp and red pulp, confirming the ability of spatially-resolved proteomics to capture important functional differences.

## Discussion

In this study, we developed and demonstrated a streamlined tandem tip-based workflow for sensitive and scalable nanoscale phosphoproteomics analysis that also integrated a front-end SOP for initial sample processing with minimal sample loss and the boosting/carrier-assisted iBASIL approach for multiplexed, sensitive, and effective MS analysis of the resulting nanoscale phosphoproteomics sample. This workflow was shown to recapitulate the major signaling pathway changes in as few as 100 mock and EGF-treated MCF10A cells and 200 µm × 200 µm × 10 µm human spleen tissue voxels.

Our home-made tandem tip-based method is a sensitive, robust and readily adoptable method for both label-free (DDA and DIA) and TMT-based nanoscale phosphoproteomics. The tandem tips can also be inserted into either Eppendorf vials or 96-well plates (via an adapter) for high-throughput phosphopeptide enrichment using centrifugation. We carefully evaluated the performance of this tandem tip-based method (e.g., sensitivity, reproducibility, and quantitation accuracy) using label-free MS analysis and low-input levels of proteins from cultured cells, demonstrating significantly improved results compared to previously reported methods/platforms. For label-free DDA analysis, the tandem tip-based method can identify 9675 (12,824 after MBR) phosphopeptides from 10 μg of proteins from A549 cells. Using DIA, the number of phosphopeptides increases to 9831 and 18,100 by direct- and library-DIA, respectively. Compared to previous studies (Supplementary Data 4), the coverage is much higher than that from the optimized AssayMAP Bravo platform using Fe^3+^-IMAC cartridges (4541 phosphopeptides without MBR from 10 μg of proteins from HeLa cells)^13^ and EasyPhos method using TiO_2_ (~4000 (~8000 after MBR) phosphopeptides from 12.5 μg of proteins from U-87 MG cells)^12^. Chen et al.^14^ further developed an integrated approach (termed Phospho-SISPROT) which used SCX/C18 tip for digestion and then transferred the digested peptides into Tip-IMAC/C18 tip. However, the phosphopeptides in the elution buffer can’t be directly loaded into C18 tip for desalting due to elution buffer contains higher organic solve (50% ACN) and higher pH salt (10% NH3·H2O). Therefore, only approximately 3000 phosphopeptides can be identified from 10 μg of HEK293T cell digests after MBR (Supplementary Data 4), respectively. This suggests that our tandem tip-based method reduces sample loss and provides higher phosphopeptide recovery (although the use of different LC and MS instruments and cell lines may still affect the final phosphoproteome coverage).

The same trend remains when the starting materials decreased to 1 μg or lower input levels (Supplementary Data 4); however, the phosphoproteome coverage became much more limited, with only ~200 phosphopeptides detected at the 0.1-μg input level by either our tandem tip method or the AssayMAP Bravo platform^13^. This is likely due to the lack of sufficient MS1 (precursor) ion intensities for triggering MS/MS in the current Orbitrap instruments. Indeed, although the IT accumulation time was set to 50 ms for MS1 data acquisition from 0.1 μg of cell lysate digests, the median of the actual IT time was only 4.7 ms (Fig. S8). This is because uninformative singly charged ions dominated the MS spectra during full m/z range acquisition in the C-trap^42–44^. This issue can be alleviated by recent developments for improving the MS1 signal in label-free analysis. For example, Meier et al. reported a BoxCar approach which was built on the ability of the quadrupole–Orbitrap mass analyzer to be filled sequentially with ions in different mass windows to increase the actual ion injection time at MS1 level^43^. High field asymmetric ion mobility spectrometry (FAIMS)^45^ is another attractive option where an asymmetric electric field was used to selectively filter ion populations by varying the compensation voltage. FAIMS has been used to selectively remove +1 ions while broadly transmitting multiply charged peptides^3,46^. Recently, Woo et al. developed an ion mobility-enhanced MS acquisition and peptide identification method, TIFF (Transferring Identification based on FAIMS Filtering), which significantly extends the ion accumulation times for peptide ions by filtering out singly charged background ions^44^. Note that a tradeoff from using high ion injection times to increase precursor ion sampling efficiency is that the cycle time is also increased significantly, which leads to substantially reduced number of MS/MS spectra for peptide identification. Therefore, the overall effectiveness of either BoxCar or TIFF approach mainly depends on matching MS1 features to an existing library via the MBR algorithm^47^. Nevertheless, integration of our tandem tip-based sample processing and enrichment method with the TIFF or BoxCar approach is still expected to increase the detection sensitivity for the label-free analysis of nanoscale phosphopeptides.

In contrast to the label-free analysis, the TMT-based boosting strategy can increase not only sample throughput (up to 18 channels)^48^ but also detection sensitivity with the use of the carrier proteome in the boosting channel for significantly improved MS1 signal. This boosting strategy has recently been demonstrated for effective single-cell proteomics analysis^1,15,17^. It has also been applied to study low abundant PTMs such as phosphotyrosine peptides^19^ or secreted glycoproteins^49^. When integrated with our streamlined tandem tip-based method, the boosting strategy enabled precise quantification of ~600 phosphopeptides from 100 sorted cells (Fig. 5) as well as >1500 phosphopeptides from 10 ng of cell lysate digests (~100 cells; Fig. 4). To increase the ion sampling from the actual low-input sample under the masking by the boosting material^24^, we applied similar iBASIL settings^17^, i.e., high AGC (1E6) and high ion injection times (0.5 or 1.5 s in Figs. 4 and 3 s in Fig. 5). To minimize the impact of TMT reagent isotopic impurities on quantitation quality^50^, especially when higher boosting ratio is used (Fig. S5), more TMT channels adjacent to the boosting channel can be kept empty (Fig. 5). In addition, the use of narrower isolation windows (0.7 Da), and the high IMAC enrichment specificity achievable for TMT-labeled phosphopeptides (~98% in Fig. 5; ~84% in Fig. 6) using the tandem tip-based method, can also effectively reduce the compression caused by the co-isolated and often relatively abundant non-phosphopeptides. Application of these approaches led to high ion purity (close to 1) for the detected phosphopeptides in 100 MCF10A cells and 100 cell-equivalent human spleen tissue voxels (Fig. S9). Additional developments, such as the use of infrared photoactivation to boost reporter ion yield in isobaric tagging^51^, are expected to further improve the performance of iBASIL-like analysis.

To better realize the potential for single-cell PTM analysis, it has been suggested that the PTM peptides enriched from bulk samples may be mixed with the un-enriched single cells for direct LC-MS/MS analysis of the PTMs^52^. This strategy may reduce the sample loss for the single-cell samples, however, the co-isolation of phosphopeptides and non-phosphopeptides in MS/MS will continue to be a major issue for quantitation (reduction in quantitation values), in addition to the masking effect by the boosting material. Indeed, phosphoproteins are often of low abundance and phosphorylation events are commonly happening at low stoichiometry^53–55^. Furthermore, the physicochemical properties of phosphopeptides critically determine their ionization efficiency^56,57^. For example, removing the phosphate group can increase the ion generation efficiency ~10-fold compared to that of the original phosphopeptides^58^, which could greatly decrease the sensitivity for detection of the signal from the single-cell phosphopeptides, especially when the single-cell phosphopeptides and non-phosphopeptides are co-isolated for MS analysis. Therefore, to achieve highly effective single-cell phosphoproteome analysis, phosphopeptide enrichment is needed and the detection sensitivity needs to be further improved. One future development will focus on improvements in LC-MS detection sensitivity by effective integration of ultralow-flow LC and a higher efficiency ion source/ion transmission interface with the most advanced MS platform and data acquisition methods. Another direction is to utilize higher resolution IMS (e.g., structures for lossless ion manipulations, SLIM^59^) for gas-phase separation to reduce sample complexity and effectively remove unwanted ions (e.g., singly charged ions, co-isolated species and isotopic impurity), resulting in increased detection sensitivity and improved quantitation accuracy. To improve the robustness and throughput, the PTM enrichment processing can also implement automated small-volume liquid handling via the use of a 96-well plate adapter/holder.

The ability to comprehensively characterize PTMs at a nanoscale or single-cell level is critical for better understanding of biological variability at functional levels. To this end, we have developed a readily implemented streamlined tandem tip-based method for nanoscale phosphoproteomics. This method capitalizes on seamless integration of C18 with IMAC to form tandem C18-IMAC-C18 for rapid, effective phosphopeptide enrichment. Further integration with our SOP and iBASIL methods forms a streamlined workflow allowing precise quantification of ~600 phosphopeptides from 100 MCF10A cells sorted by FACS and ~700 phosphopeptides from 200 µm × 200 µm × 10 µm human spleen tissue voxels (equivalent to ~100 cells). Moreover, the resulting high-quality phosphoproteome data were able to capture important functional differences in the samples. This streamlined workflow should open new avenues for nanoscale phosphoproteome profiling of small numbers of cells and mass-limited samples at the low nanogram levels and high-resolution spatial phosphoproteome mapping of human tissues, which cannot be accessed by current proteomics platforms. With further improvements in detection sensitivity as well as sample processing, it has the potential to moving towards single-cell phosphoproteomics.

## Methods

### Reagents

Urea, dithiothreitol (DTT), iodoacetamide (IAA), iron chloride, triethylammonium bicarbonate (TEABC), ethylenediaminetetraacetic acid (EDTA), ammonia phosphate (NH_4_H_2_PO_4_), trifluoroacetic acid (TFA), formic acid (FA), acetonitrile (ACN), n-Dodecyl β-D-maltoside (DDM), protease, phosphatase inhibitor, DMSO (HPLC grade), and Phosphate-Buffered Saline (PBS) were obtained from Sigma (St. Louis, MO). The Ni-NTA silica beads and agarose beads were obtained from Qiagen (Hilden, Germany) and the Empore^TM^ extraction C18 disks were obtained from 3 M (St. Paul, MN). TMT-16 reagents were purchased from Thermo Fisher Scientific (Waltham, MA). Water was processed using a Millipore Milli-Q system (Bedford, MA). Polypropylene microwell chip with 2.2-mm wells diameter was manufactured on polypropylene substrates by Protolabs (Maple Plain, MN). Tris(2-carboxyethyl) phosphine hydrochloride (TCEP-HCl), and 50% Hydroxylamine (HA) were purchased from Thermo Fisher Scientific (Waltham, MA). Ethanol was purchased from Decon Labs, Inc. (King of Prussia, PA).

### Cell culture and protein digestion

The AML (MOLM-14, K562 and CMK), MCF-7, A549 and MCF10A breast cell lines were obtained from the American Type Culture Collection and were prepared as previously described^60^. For EGF stimulation, EGF (PeproTech, Rocky Hill, NJ) was added directly to the MCF10A cell plates at 10 ng/mL and cultured for 30 min before rinse and harvest. Cell pellets were washed with ice-cold phosphate-buffered saline (PBS), lysed in a lysis buffer containing 50 mM TEABC, pH 8.0, 8 M urea, and a 1% protease and phosphatase inhibitor. The protein concentration was determined using the BCA protein assay (Thermo Fisher Scientific). The protein solutions were denatured with 10 mM DTT for 15 mins at 37 °C and alkylated with 50 mM iodoacetamide in the dark for 30 mins at room temperature. The resulting samples were diluted by 8-fold with 50 mM TEABC and digested by lysyl endopeptidase and trypsin (protein:enzyme, 20:1, w/w) at 37 °C overnight. The digested tryptic peptides were acidified by TFA with a final TFA concentration of 0.5%, and then desalted by C18 SPE extraction and concentrated for BCA assay.

### TMT labeling for the bulk samples

For bulk digestion peptides, the digested peptides (in 200 mM HEPES) were mixed with TMT-16 reagents dissolved in 100% ACN and allowed to react for 1 h. An optimized ratio of TMT to peptide amount of 1:1 (w/w)^61^ was used (i.e., 100 μg of peptides labeled by 100 μg of TMT reagent). The labeling reactions were stopped by adding 5% hydroxylamine (final concentration is 0.5%) for 15 min and then acidified with TFA (final concentration is 0.5%). Peptides labeled with different TMT tags were mixed in the same tube, after which the ACN concentration was adjusted to below 5% (v/v) and the samples were desalted by C18 SPE.

### FACS sorting and protein digestion

For procedure of cell sorting has been descripted in the previous study^20^. The sorted cells were denatured with 1 μL 0.2% DDM with 10 mM TCEP in 50 mM TEABC. Samples were incubated at 75 °C for 1 h for denaturation and reduction. After that, the samples were alkylated with 50 mM IAA in the dark for 30 mins at room temperature. The resulting samples were digested by 10 ng lysyl endopeptidase and 10 ng trypsin at 37 °C overnight (The final volume was 3 μL). The digested tryptic peptides were diluted with 1 μL 200 mM HEPES (pH 8.8) labeled with 1 μL TMTpro reagent (20 μg/μL) for 1 h at room temperature. Then, the labeling peptides were treated with 0.25 μL 5% hydroxylamine for 15 mins and acidified by 1.25 μL 10% TFA and 1.25 μL 10% FA. Peptides labeled with different TMT tags were mixed in the same tube, after which the ACN concentration was adjusted to below 5% (v/v) and the samples were loaded into tandem tip C18-IMAC-C18 for phosphopeptide enrichment/cleanup.

### LCM of tissue sections and protein digestion

The spleen tissue from a 33-years old Caucasian male was collected as organ donor under informed consent of the Human Biomolecular Atlas Program of the National Institutes of Health Common Fund, which received ethical approval, and the study protocol (IRB201600029) was approved by the University of Florida Institutional Review Board. Frozen spleen tissue sample was mounted on a cryomicrotome chuck by freezing a small droplet of water, and then sectioned using a blade temperature of −16 °C and specimen temperature of −23 °C. Ten-micrometer-thick sections were thaw-mounted onto polyethylene naphthalate (PEN) membrane slides (Zeiss) for subsequent laser capture microdissection analysis. Cryosectioning was performed using CryoStar NX70, Thermo Fisher.

The tissue section was washed and dehydrated by immersing slide in the gradient of ethanol solutions for 30 s each change (70% Ethanol, 95% Ethanol, and 100% Ethanol, respectively). Two distinct areas of the spleen tissue (white and red pulp) were dissected and collected in the microwell chip using PALM MicroBeam laser capture microdissection system (Carl Zeiss MicroImaging, Munich, Germany). Microwells were preloaded with 3 µl of DMSO, which served as a capturing medium for the excised tissue sections. Four areas of white pulp and four areas of red pulp (each area 200 µ × 200 µm) were dissected and collected in the corresponding wells of the microPOTS chip. A two-million-square-micrometer pool of white and red pulps was collected in the single microwell, which was used as a boost to increase the MS detection sensitivity.

MicroPOTS chip and its cover were incubated at 75 °C for an hour to dry DMSO solvent. Next, 2 µL of extraction buffer containing 0.1% DDM, 0.5×PBS, 38 mM TEAB, and 1 mM TCEP was dispensed into each well of the chip. The chip was incubated at 75 °C for an hour. 0.5 µL of IAA solution (10 mM IAA in 100 mM TEAB) was added to the corresponding wells with the samples, following the incubation step at room temperature for 30 min. All samples were subsequently digested by adding 0.5 µL of an enzyme mixture (10 ng of Lys-C and 40 ng of trypsin in 100 mM TEAB). Enzymes’ quantities were doubled for the boosting sample. After incubation at 37 °C for 10 h, peptides were labeled with TMTpro 16plex by adding 1 µL of TMT isobaric mass tag reagent (resuspended in anhydrous acetonitrile to a concentration of 5 μg/μL) to the corresponding well with the sample. Samples were labeled at room temperature for an hour. Following labeling, samples were quenched by 1 µL of 5% hydroxylamine in 100 mM TEAB and incubated at room temperature for 15 min. Next, samples were pooled together, brought up to the final 1% FA, then centrifuged at 10,000 rpm for 5 min at 25 °C. The pooled sample was then transferred to an autosampler vial, coated with 0.01% DDM, and dried.

### Phosphopeptide enrichment by IMAC

For agarose beads, the phosphopeptides enrichment was prepared as previously described^10^. For silica beads, the design for sensitivity improvement was based on an old version of IMAC tip^57^. The in-house-made IMAC tip was capped in a tip-end with a 20-μm polypropylene frit disk followed by packing with Ni-NTA silica resin. The IMAC tip was inserted into 1.5 mL Eppendorf or 96 well tip holder. First, Ni^2+^ ions were removed by adding 50 mM EDTA in 1 M NaCl (1000 g, 1 min). The tip was then activated with 100 mM FeCl_3_ (1000 g, 1 min) and equilibrated with 1% (v/v) acetic acid (1000 g, 1 min) at pH 3.0, prior to sample loading. For IMAC-C18 workflow, tryptic peptides were dissolved in 0.1% (v/v) TFA, 80% ACN and loaded onto the IMAC tip (1000 g, 1 min). For C18-IMAC-C18 workflow, the C18 tips which contain tryptic peptides were inserted into activated IMAC tips and the peptides were eluted from C18 tip to IMAC tip by 0.1% (v/v) TFA, 80% ACN (1000 g, 3 min). Then, the IMAC tip were washed by using 1% (v/v) TFA, 80% ACN (1000 g, 1 min), and 1% (v/v) acetic acid (1000 g, 1 min), respectively. The IMAC tip was then inserted into activated desalting C18 StageTip. The bound phosphopeptides were eluted by 200 mM NH_4_H_2_PO_4_ (1000 g, 3 min) onto the activated desalting C18 StageTip for desalting and directly eluted to sample vials of LC-MS, then dried under vacuum. For SRM analysis, 1 μL of 4000 fmol/μL of each crude SIL phosphopeptide was spiked into 200 μL of 1 μg/μL tryptic peptides before IMAC enrichment.

### Phosphopeptide enrichment and in-Tip high-pH reversed-phase StageTip

After phosphopeptides enrichment, the IMAC tip was directly inserted into the activate High-pH C18 StageTip that was packed with 5-µm C18 beads (Dr. Maisch-GmbH)^21^ before elution. The phosphopeptides were directly eluted from the IMAC tip into the activate High-pH C18 StageTip. Then, the phosphopeptides were fractionated with increasing concentration of ACN (see Supplementary Data 5) and concatenated into 4 or 6 fractions. Eluted phosphopeptides were dried with SpeedVac and stored at −80 °C until LC-MS/MS analysis.

### Stable isotope-labeled peptides

Ten crude stable isotope-labeled (SIL) phosphopeptides were synthesized with 13 C/15 N on C-terminal lysine or arginine (New England Peptide, Gardner, MA). The SIL peptides were dissolved in 15% ACN and 0.1% FA at a concentration of 1.5 mM individually. A mixture of SIL peptides was made with a final concentration of 4 pmol/μL for each peptide.

### LC-MS/MS Analysis

For most of experiments, lyophilized peptides were reconstituted in 12 μL 0.1% TFA with 2% ACN containing 0.01% DDM^20^ and 5 μL of the resulting sample was analyzed by LC-MS/MS using an Orbitrap Fusion Lumos Tribrid mass spectrometer (Thermo Scientific) connected to a nanoACQUITY UPLC system (Waters) (buffer A: 0.1% FA with 3% ACN and buffer B: 0.1% FA in 90% ACN) as previously described^17^. Peptides were separated by a gradient mixture with an analytical column (75 μm i.d. × 20 cm) packed using 1.9-μm ReproSil C18 and with a column heater set at 50 °C. Peptides were separated by an LC gradient: 2–6% buffer B in 1 min, 6–30% buffer B in 84 min, 30–60% buffer B in 9 min, 60–90% buffer B in 1 min, and finally 90% buffer B for 5 min at 200 nL/min. For experiments in Fig. 6, lyophilized peptides were reconstituted in 0.1% TFA with 2% ACN containing 0.01% DDM and injected using a PAL autosampler (CTC Analytics AG, Switzerland). The sample was concentrated into an online SPE column (150 μm i.d., 360 μm o.d., 4 cm long) and separated using a 50 μm i.d., 360 μm o.d., 50 cm long column packed with 3-μm C18 particles (300-Å pore size; Phenomenex, Terrence, CA). The nanoLC separation used a Dionex UltiMate NCP-3200RS system (Thermo Scientific, Waltham, MA) with mobile phases of water with 0.1% FA (buffer A) and ACN with 0.1% FA (buffer B). Peptides were separated through a linear gradient from 8% to 35% buffer B over 100 min at a flow rate of 150 nL/min. The separated peptides were analyzed using a Thermo Scientific Q Exactive Plus. Top 10 precursor ions were selected for MS/MS. Different AGC settings and maximum ITs at MS and MS/MS level for different DDA experiments were listed in Supplementary Data 1. The DIA-MS/MS scan was performed in the HCD mode with the following parameters: precursor ions from 350–1650 *m/z* were scanned at 120,000 resolution with an ion injection time of 60 ms and an AGC target of 3E6. The scan rage of m/z (isolation window) of DIA windows from 377 (54), 419(32), 448(28), 473.5(25), 497.5(25), 520.5 (23), 542.5 (23), 564.5 (23), 587 (24), 610.5 (25), 635 (26), 660 (26), 685.5 (27), 712.5 (29), 741 (30), 771 (32), 803.5 (35), 838.5 (37), 877 (42), 921 (48), 972 (52), 1034.5 (71), 1133.5 (129) and 1423.5 (453) were scanned at 15,000 resolution with an ion injection time of 120 ms and an AGC target of 3E6. The isolated ions were fragmented with HCD at 30% level.

### SRM assay development and LC-SRM

To evaluate the peptide quality and select the best transitions for each peptide, heavy peptide mixtures were subjected to an initial analysis using a TSQ Altis triple Quadrupole Mass Spectrometer (Thermo Fisher Scientific) equipped with a nanoACQUITY UPLC system (Waters, Milford, MA). The collision energies for individual transitions were obtained by using empirical equations from the Skyline software. The 6 most intensive fragment ions for each peptide were initially selected. Next, the LC characteristics, MS response, interferences, and endogenous detectability of the SIL peptides were further evaluated by spiking them into water and pooled samples for IMAC enrichment and LC-SRM analysis. In the end, 3 or more transitions per peptide were selected for configuration of the final assays for reproducible targeted quantification. The eluent from IMAC was dissolved in 10 µL 0.1% TFA with 2% ACN containing 0.01% DDM and 9 μL of the resulting sample was loaded onto the LC column (100 µm i.d. × 10 cm, BEH 1.7-µm C18 capillary column (Waters)) and separated under the following conditions: mobile phases (A) 0.1% FA in water and (B) 0.1% FA in ACN; flow rate at 400 nL/min; 42-min gradient (min:%B): 11:0.5, 13: 5, 18:20, 23:25, 29:40, 31:95, 36:0.5. The LC column was operated at a temperature of 44 ^o^C. The parameters of the triple quadruple instrument were set as follows: 0.7 fwhm Q1 and Q3 resolution, and 1 s cycle time. Data were acquired in the unscheduled SRM without blinding the samples.

### Data analysis for DDA experiment

The raw MS/MS data were processed with MSFragger via FragPipe^62,63^. The MS/MS spectra were searched against a human UniProt database (fasta file dated July 31, 2021 with 40,840 sequences which contain 20,420 decoys) and (initial) fragment mass tolerances were set to 20 ppm. A peptide search was performed with full tryptic digestion (Trypsin) and allowed a maximum of two missed cleavages. Carbamidomethyl (C) was set as a fixed modification; acetylation (protein N-term), oxidation (M) and Phospho (STY) were set as variable modifications for global proteome analysis. For match-between-run (MBR) analysis, 10 ppm m/z tolerance, 0.4 mins RT tolerance and 0.05 MBR ions FDR were used for analysis. The final reports were then generated (PSM, ion, peptide, and protein) and filtered at each level (1% protein FDR plus 1% PSM/ion/peptide-level FDR for each corresponding PSM.tsv, ion.tsv, and peptide.tsv files). For TMT experiments, TMTpro labeling (K) and (peptide N-term) was set as a fixed modification. The intensities of all ten TMT reporter ions were extracted from Fragpipe outputs and analyzed by Perseus^64^ for statistical analyses.

### Data analysis for DIA experiment

The DIA data analysis was performed essentially as described in the DDA data analysis (above), except that a library file was further imported into DIA-NN^65^ for DIA library matching.

### Data analysis for SRM experiment

SRM data were analyzed using Skyline software (version 4.2). The total peak area ratios of endogenous light peptides and their heavy isotope-labeled internal standards (i.e., L/H peak area ratios) were calculated for quantification. Peak detection and integration were carried out based on two criteria: (1) same retention time and (2) similar relative SRM peak intensity ratios across multiple transitions between light (endogenous) peptides and the heavy SIL peptide standards. All data were manually inspected to ensure correct peak detection and accurate integration.

### Statistics and Reproducibility

For phosphoproteome analysis, at least three biological replicates were collected for each data; each biological replicate is treated as one sample during data analysis. No data exclusion was performed, and no randomization or blinding methods were used in data analysis. The extracted TMT reporter ion intensity of the identified phosphopeptides was log2 transformed, and then normalized using the median centering approach. The quantitation values for each phosphorylation sites containing at least 70% no-missing values in one group were kept in the data matrix; in cases where the missing values were imputed, the normal distribution approach with a width of 0.3 and a downshift of 1.8 was used with Perseus^64^. The non-supervised PCA analysis was used to generate PCA plot. ANOVA *t* test was used to prioritize significantly differentiated phosphorylation sites (*p* < 0.05, FDR < 0.2) for generating the volcano plot.

### CODEX Staining and Imaging

Barcoded antibody staining of tissue sections mounted on cover slips was performed using a commercially available CODEX Staining Kit according to the manufacturer’s instructions. Raw images were processed and stitched using CODEX Processor software including cycle alignment, drift compensation, background subtraction, and cell segmentation. Image analysis was performed with the Akoya Multiplex Analysis Viewer (MAV) in Fiji with KNN/FLANN clustering, gating and spatial network mapping.

### Reporting summary

Further information on research design is available in the Nature Portfolio Reporting Summary linked to this article.

## Supplementary information

Supplementary Information

Description of Additional Supplementary Files

Supplementary Data 1

Supplementary Data 2

Supplementary Data 3

Supplementary Data 4

Supplementary Data 5

Reporting Summary

## Data Availability

The RAW global MS data and the identified results from Fragpipe have been deposited in Japan ProteOme STandard Repository (jPOST: https://repository.jpostdb.org/)^66^. The accession codes: JPST001468 for jPOST and PXD032019 for ProteomeXchange. The access link is https://repository.jpostdb.org/preview/189170729262298fbf5daee and access key is 4830. SRM results including the assay characterization data are organized as Skyline files on the Panorama server^67^ and can be accessed via https://panoramaweb.org/nanoscale_phosphoproteomics.url (Email: panorama+reviewer108@proteinms.net; Password: oHgxvRzH). The index of all the source data was listed in Supplementary Data 1.

## Code availability

No custom code was used to generate or process the data described in this work.

## References

[1] Woo J (2021). Author correction: high-throughput and high-efficiency sample preparation for single-cell proteomics using a nested nanowell chip. Nat. Commun..

[2] Specht H (2021). Single-cell proteomic and transcriptomic analysis of macrophage heterogeneity using SCoPE2. Genome Biol..

[3] Cong Y (2020). Ultrasensitive single-cell proteomics workflow identifies >1000 protein groups per mammalian cell. Chem. Sci..

[4] Schoof EM (2021). Quantitative single-cell proteomics as a tool to characterize cellular hierarchies. Nat. Commun..

[5] Polat AN, Ozlu N (2014). Towards single-cell LC-MS phosphoproteomics. Analyst..

[6] Huttlin EL (2010). A tissue-specific atlas of mouse protein phosphorylation and expression. Cell..

[7] Lundby A (2012). Quantitative maps of protein phosphorylation sites across 14 different rat organs and tissues. Nat. Commun..

[8] Bekker-Jensen DB (2017). An optimized shotgun strategy for the rapid generation of comprehensive human proteomes. Cell Syst..

[9] Hogrebe A (2018). Benchmarking common quantification strategies for large-scale phosphoproteomics. Nat. Commun..

[10] Mertins P (2018). Reproducible workflow for multiplexed deep-scale proteome and phosphoproteome analysis of tumor tissues by liquid chromatography-mass spectrometry. Nat. Protoc..

[11] Humphrey SJ, Azimifar SB, Mann M (2015). High-throughput phosphoproteomics reveals in vivo insulin signaling dynamics. Nat. Biotechnol..

[12] Humphrey SJ, Karayel O, James DE, Mann M (2018). High-throughput and high-sensitivity phosphoproteomics with the EasyPhos platform. Nat. Protoc..

[13] Post H (2017). Robust, sensitive, and automated phosphopeptide enrichment optimized for low sample amounts applied to primary hippocampal neurons. J. Proteome Res..

[14] Chen W, Chen L, Tian R (2018). An integrated strategy for highly sensitive phosphoproteome analysis from low micrograms of protein samples. Analyst..

[15] Budnik B, Levy E, Harmange G, Slavov N (2018). SCoPE-MS: mass spectrometry of single mammalian cells quantifies proteome heterogeneity during cell differentiation. Genome Biol..

[16] Dou M (2019). High-throughput single cell proteomics enabled by multiplex isobaric labeling in a nanodroplet sample preparation platform. Anal. Chem..

[17] Tsai CF (2020). An improved boosting to amplify signal with isobaric labeling (iBASIL) strategy for precise quantitative single-cell proteomics. Mol. Cell Proteom..

[18] Yi L (2019). Boosting to amplify signal with isobaric labeling (BASIL) strategy for comprehensive quantitative phosphoproteomic characterization of small populations of cells. Anal. Chem..

[19] Chua XY (2020). Tandem mass tag approach utilizing pervanadate boost channels delivers deeper quantitative characterization of the tyrosine phosphoproteome. Mol. Cell Proteom..

[20] Tsai CF (2021). Surfactant-assisted one-pot sample preparation for label-free single-cell proteomics. Commun. Biol..

[21] Dimayacyac-Esleta BR (2015). Rapid high-ph reverse phase stagetip for sensitive small-scale membrane proteomic profiling. Anal. Chem..

[22] Zhu Y (2018). Spatially resolved proteome mapping of laser capture microdissected tissue with automated sample transfer to nanodroplets. Mol. Cell Proteom..

[23] Dou M (2019). Automated nanoflow two-dimensional reversed-phase liquid chromatography system enables in-depth proteome and phosphoproteome profiling of nanoscale samples. Anal. Chem..

[24] Cheung TK (2021). Defining the carrier proteome limit for single-cell proteomics. Nat. Methods..

[25] Ye Z, Batth TS, Ruther P, Olsen JV (2022). A deeper look at carrier proteome effects for single-cell proteomics. Commun. Biol..

[26] Huang da W, Sherman BT, Lempicki RA (2009). Systematic and integrative analysis of large gene lists using DAVID bioinformatics resources. Nat. Protoc..

[27] Szklarczyk D (2019). STRING v11: protein-protein association networks with increased coverage, supporting functional discovery in genome-wide experimental datasets. Nucleic Acids Res..

[28] Heisermann GJ (1990). Mutational removal of the Thr669 and Ser671 phosphorylation sites alters substrate specificity and ligand-induced internalization of the epidermal growth factor receptor. J. Biol. Chem..

[29] Patrussi L (2005). Cooperation and selectivity of the two Grb2 binding sites of p52Shc in T-cell antigen receptor signaling to Ras family GTPases and Myc-dependent survival. Oncogene..

[30] Theard D, Raspe MA, Kalicharan D, Hoekstra D, van ISC (2008). Formation of E-cadherin/beta-catenin-based adherens junctions in hepatocytes requires serine-10 in p27(Kip1). Mol. Biol. Cell..

[31] Coluccia AM (2007). Bcr-Abl stabilizes beta-catenin in chronic myeloid leukemia through its tyrosine phosphorylation. EMBO J..

[32] Sato S, Fujita N, Tsuruo T (2002). Regulation of kinase activity of 3-phosphoinositide-dependent protein kinase-1 by binding to 14-3-3. J. Biol. Chem..

[33] Jurek A, Amagasaki K, Gembarska A, Heldin CH, Lennartsson J (2009). Negative and positive regulation of MAPK phosphatase 3 controls platelet-derived growth factor-induced Erk activation. J. Biol. Chem..

[34] Smith RG, Reynolds CP (1987). Monoclonal antibody recognizing a human neuroblastoma-associated antigen. Diagn. Clin. Immunol..

[35] Bolnick J, Albitar L, Laidler LL, Abdullah R, Leslie KK (2011). Blocking Epidermal Growth Factor Receptor Signaling in HTR-8/SVneo First Trimester Trophoblast Cells Results in Dephosphorylation of PKBalpha/AKT and Induces Apoptosis. Obstet. Gynecol. Int..

[36] Zhou Z (2012). The Akt-SRPK-SR axis constitutes a major pathway in transducing EGF signaling to regulate alternative splicing in the nucleus. Mol. Cell..

[37] Xu K (2019). Benchtop-compatible sample processing workflow for proteome profiling of <100 mammalian cells. Anal. Bioanal. Chem..

[38] Ogembo JG (2012). SIRPalpha/CD172a and FHOD1 are unique markers of littoral cells, a recently evolved major cell population of red pulp of human spleen. J. Immunol..

[39] Lewis, S. M., Williams, A. & Eisenbarth, S. C. Structure and function of the immune system in the spleen. *Sci. Immunol*. **4**, eaau6085 (2019). 10.1126/sciimmunol.aau6085 PMC6495537 30824527

[40] Klasener, K. et al. CD20 as a gatekeeper of the resting state of human B cells. *Proc. Natl. Acad. Sci. USA* **118**, e2021342118 (2021). 10.1073/pnas.2021342118 PMC7896350 33563755

[41] Jongstra-Bilen J, Wielowieyski A, Misener V, Jongstra J (1999). LSP1 regulates anti-IgM induced apoptosis in WEHI-231 cells and normal immature B-cells. Mol. Immunol..

[42] Loo JA, Udseth HR, Smith RD (1989). Peptide and protein analysis by electrospray ionization-mass spectrometry and capillary electrophoresis-mass spectrometry. Anal. Biochem..

[43] Meier F, Geyer PE, Virreira Winter S, Cox J, Mann M (2018). BoxCar acquisition method enables single-shot proteomics at a depth of 10,000 proteins in 100 min. Nat. Methods..

[44] Woo, J. et al. Three-dimensional feature matching improves coverage for single-cell proteomics based on ion mobility filtering. *Cell Syst.* **13**, 426–434.e4. (2022). 10.1016/j.cels.2022.02.003 PMC9119937 35298923

[45] Guevremont R (2004). High-field asymmetric waveform ion mobility spectrometry: a new tool for mass spectrometry. J. Chromatogr. A..

[46] Bekker-Jensen DB (2020). A compact quadrupole-orbitrap mass spectrometer with FAIMS interface improves proteome coverage in short LC gradients. Mol. Cell. Proteom..

[47] Tyanova S, Temu T, Cox J (2016). The MaxQuant computational platform for mass spectrometry-based shotgun proteomics. Nat. Protoc..

[48] Li J (2021). TMTpro-18plex: the expanded and complete set of TMTpro reagents for sample multiplexing. J. Proteome Res..

[49] Suttapitugsakul S, Tong M, Sun F, Wu R (2021). Enhancing comprehensive analysis of secreted glycoproteins from cultured cells without serum starvation. Anal. Chem..

[50] Searle BC, Yergey AL (2020). An efficient solution for resolving iTRAQ and TMT channel cross-talk. J. Mass Spectrom..

[51] Lee KW (2022). Infrared photoactivation boosts reporter ion yield in isobaric tagging. Anal. Chem..

[52] Kwon Y (2022). Phosphoproteome profiling using an isobaric carrier without the need for phosphoenrichment. Anal. Chem..

[53] Tsai CF (2015). Large-scale determination of absolute phosphorylation stoichiometries in human cells by motif-targeting quantitative proteomics. Nat. Commun..

[54] Olsen JV (2010). Quantitative phosphoproteomics reveals widespread full phosphorylation site occupancy during mitosis. Sci. Signal..

[55] Wu R (2011). A large-scale method to measure absolute protein phosphorylation stoichiometries. Nat. Methods..

[56] Zhou H (2011). Enhancing the identification of phosphopeptides from putative basophilic kinase substrates using Ti (IV) based IMAC enrichment. Mol. Cell Proteom..

[57] Tsai CF (2014). Sequential phosphoproteomic enrichment through complementary metal-directed immobilized metal ion affinity chromatography. Anal. Chem..

[58] Dreier RF, Ahrne E, Broz P, Schmidt A (2019). Global ion suppression limits the potential of mass spectrometry based phosphoproteomics. J. Proteome Res.

[59] Chouinard CD (2018). Improved sensitivity and separations for phosphopeptides using online liquid chromotography coupled with structures for lossless ion manipulations ion mobility-mass spectrometry. Anal. Chem..

[60] Shi T (2016). Conservation of protein abundance patterns reveals the regulatory architecture of the EGFR-MAPK pathway. Sci. Signal..

[61] Zecha J (2019). TMT labeling for the masses: a robust and cost-efficient, in-solution labeling approach. Mol. Cell Proteom..

[62] Kong AT, Leprevost FV, Avtonomov DM, Mellacheruvu D, Nesvizhskii AI (2017). MSFragger: ultrafast and comprehensive peptide identification in mass spectrometry-based proteomics. Nat. Methods..

[63] Teo GC, Polasky DA, Yu F, Nesvizhskii AI (2021). Fast deisotoping algorithm and its implementation in the MSFragger search engine. J. Proteome Res..

[64] Tyanova S (2016). The Perseus computational platform for comprehensive analysis of (prote)omics data. Nat. Methods..

[65] Demichev V, Messner CB, Vernardis SI, Lilley KS, Ralser M (2020). DIA-NN: neural networks and interference correction enable deep proteome coverage in high throughput. Nat. Methods..

[66] Watanabe Y, Yoshizawa AC, Ishihama Y, Okuda S (2021). The jPOST repository as a public data repository for shotgun proteomics. Methods Mol. Biol..

[67] Sharma V (2018). Panorama public: a public repository for quantitative data sets processed in skyline. Mol. Cell Proteom..

