# Supplementary material for: A streamlined tandem tip-based workflow for sensitive nanoscale phosphoproteomics
Source: Commun Biol. 2023 Jan 18;6:70. doi: 10.1038/s42003-022-04400-x (PMC9849344; doi:10.1038/s42003-022-04400-x)
Supplement: Supplementary file 1 — Supplementary Information [file 42003_2022_4400_MOESM1_ESM.pdf]

Supporting Information

A streamlined tandem tip-based workflow for sensitive nanoscale phosphoproteomics

Chia-Feng Tsai[1,#,¶], Yi-Ting Wang[1,#], Chuan-Chih Hsu[2], Reta Birhanu Kitata[1], Rosalie K. Chu[1], Marija Velickovic[3], Rui Zhao[1], Sara M. Williams[3], William B. Chrisler[1], Marda L. Jorgensen[4], Ronald J. Moore[1], Ying Zhu[3], Karin D. Rodland[1], Richard D. Smith[1], Clive H. Wasserfall[4], Tujin Shi[1¶], and Tao Liu[1¶]

[1]Biological Sciences Division, Pacific Northwest National Laboratory, Richland, WA 99354, USA
[2]Institute of Plant and Microbial Biology, Academia Sinica, Taipei, Taiwan
[3]Environmental Molecular Sciences Laboratory, Pacific Northwest National Laboratory, Richland, Washington 99354, USA
[4]Department of Pathology, Immunology, and Laboratory Medicine, Diabetes Institute, College of Medicine, University of Florida, Gainesville, Florida 32611, USA
[#]These authors contributed equally

Keywords: Tip IMAC, Boosting to Amplify the Signal with Isobaric Labeling (BASIL), near single-cell, nanoscale phosphoproteome, Surfactant-assisted One-Pot (SOP) sample preparation, spatial phosphoproteome mapping

[¶]Corresponding author:
Dr. Chia-Feng Tsai
Biological Sciences Division
Pacific Northwest National Laboratory
Richland, WA 99354
Tel: (509) 375-2924
Email: chia-feng.tsai@pnnl.gov

Dr. Tujin Shi
Biological Sciences Division
Pacific Northwest National Laboratory
Richland, WA 99354
Tel: (509) 371-6579
Email: tujin.shi@pnnl.gov

Dr. Tao Liu
Biological Sciences Division
Pacific Northwest National Laboratory
Richland, WA 99354
Tel: (509) 371-6346
Email: tao.liu@pnnl.gov

SI Figures:

Figure S1. The comparison of the step-by-step and integrated tip-based IMAC methods for phosphopeptide enrichment. (a) Schematic illustration of the two methods. The number of identified phosphopeptides/proteins, XIC area of phosphopeptides (b), Pearson correlation, and CV (%) (c) of identified phosphopeptides purified from 10 μg of proteins from the A549 cell lysate. The error bars represent the standard deviation (n=3).

Figure S2. The reproducibility of integrated tip-IMAC for phosphopeptide enrichment. Pearson correlation and CV (%) of peak areas of 10 spiked SIL phosphopeptides (SRM data) in a reference endometrial tumor sample enriched with tip-based IMAC in different days and batches.

Figure S3. The IMAC-HpH-RP tip method for microscale phosphopeptide fractionation. (a) Using the integrated C18-IMAC-HpH-RP tip method for phosphopeptide fractionation (4 fractions for 20 μg tryptic peptides of MCF-7 cells). (b) Low overlap of the phosphopeptides identified from the 4 different fractions. (c) The tandem IMAC-HpH-C18 tip for phosphopeptide fractionation (6 fractions for 500 μg of tryptic peptides of MCF-7 cells). (d) Separation efficiency calculated by the percentage of common phosphopeptides between fractions.

Figure S4. Pathway enrichment analysis of significantly changed phosphopeptides of 10 ng tryptic digests of AML cells under different ion injection times.

Figure S5. Effect of ion injection time on quantitation quality in the mimic nanoscale phosphoproteome analysis. The PCA analysis of quantified phosphopeptides of 1 ng tryptic digests of the AML cells under two different ion injection times (0.5 and 1.5 s).

Figure S6. Phosphoproteome analysis for 10 sorted MCF10A cells. (a) The numbers of quantified (70% no-missing value in study samples) phosphopeptides and enrichment specificity in each TMT experiment. (b) PCA analysis shows the clustering of cells from the two different treatment conditions.

Figure S7. Global proteome analysis of LCM-dissected human spleen tissue voxels. (a) PCA of the proteome data. (b) Volcano plot shows significantly changed proteins in white pulp and red pulp (t-test, n=8 for each condition; s0=1 and FDR=0.05% were used as cut-off values). (c) The altered protein expression of surface markers. (d) Representative CODEX image of human spleen tissue: CD8α (red), CD163 (yellow), CD3e (white), CD20 (green), CD31 (blue). The image size is 560 μm x 560 μm as shown.

Figure S8. The ion injection time distribution of purified phosphopeptides from 0.1 μg proteins from the A549 cell lysate at MS1 and MS2 level.

Figure S9. The ion purity distribution of detected phosphopeptides in Figures 6 and 7.

Supplementary Data 1. The raw files and corresponding samples, experimental conditions and MS instrument setting.

Supplementary Data 2. Pathway enrichment analysis of significantly changed phosphopeptides between EGF- and mock-treated cells. Identification results in Exp. A (a) and B (b). (c) Quantitation results. (d) KEGG pathway. (e) Reactome pathway.

Supplementary Data 3. Pathway enrichment analysis of significantly changed phosphopeptides. (a) Identification results. (b) Quantitation results. (c) Pathways enriched in red pulp. (d) Pathways enriched in white pulp.

Supplementary Data 4. The summary of the number of identified phosphopeptides in this study and other two published works.

Supplementary Data 5. Buffer composition for in-tip high-pH fractionation and concatenation.

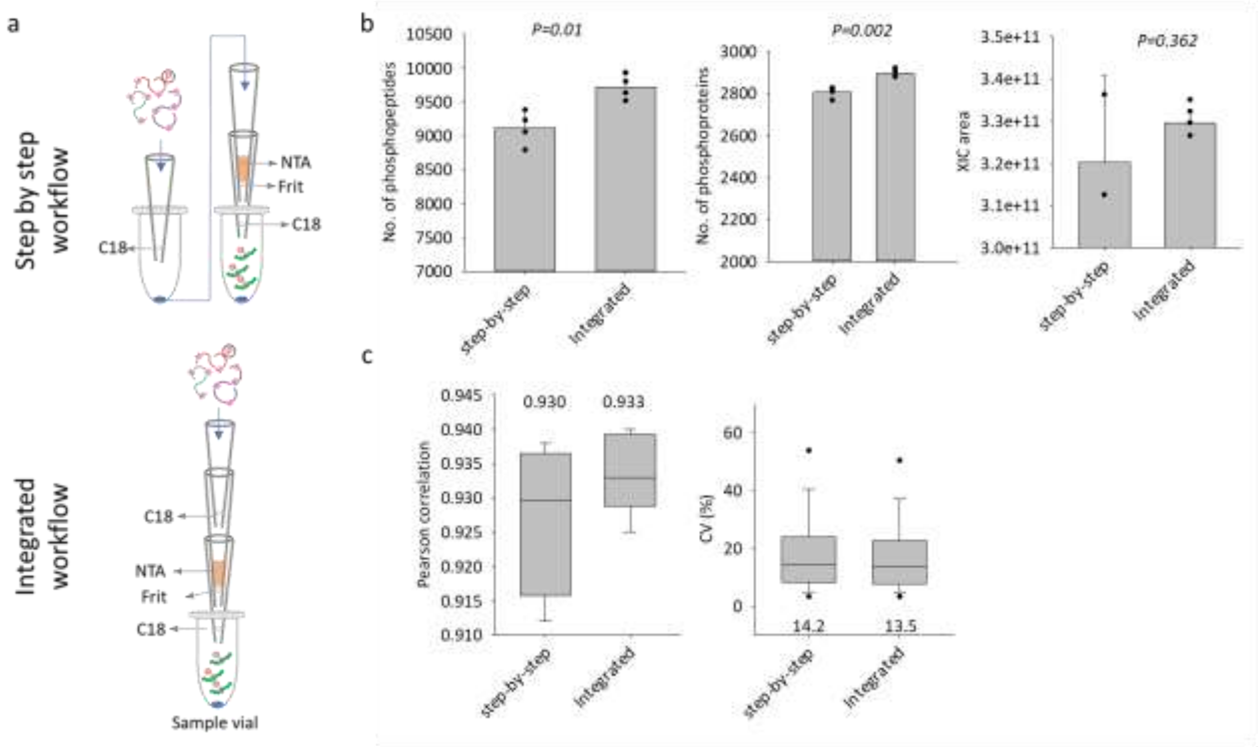

Figure S1. The comparison of the step-by-step and integrated tip-based IMAC methods for phosphopeptide enrichment. (a) Schematic illustration of the two methods. The number of identified phosphopeptides/proteins, XIC area of phosphopeptides (b), Pearson correlation, and CV (%) (c) of identified phosphopeptides purified from 10 μg of proteins from the A549 cell lysate. The error bars represent the standard deviation (n=3).

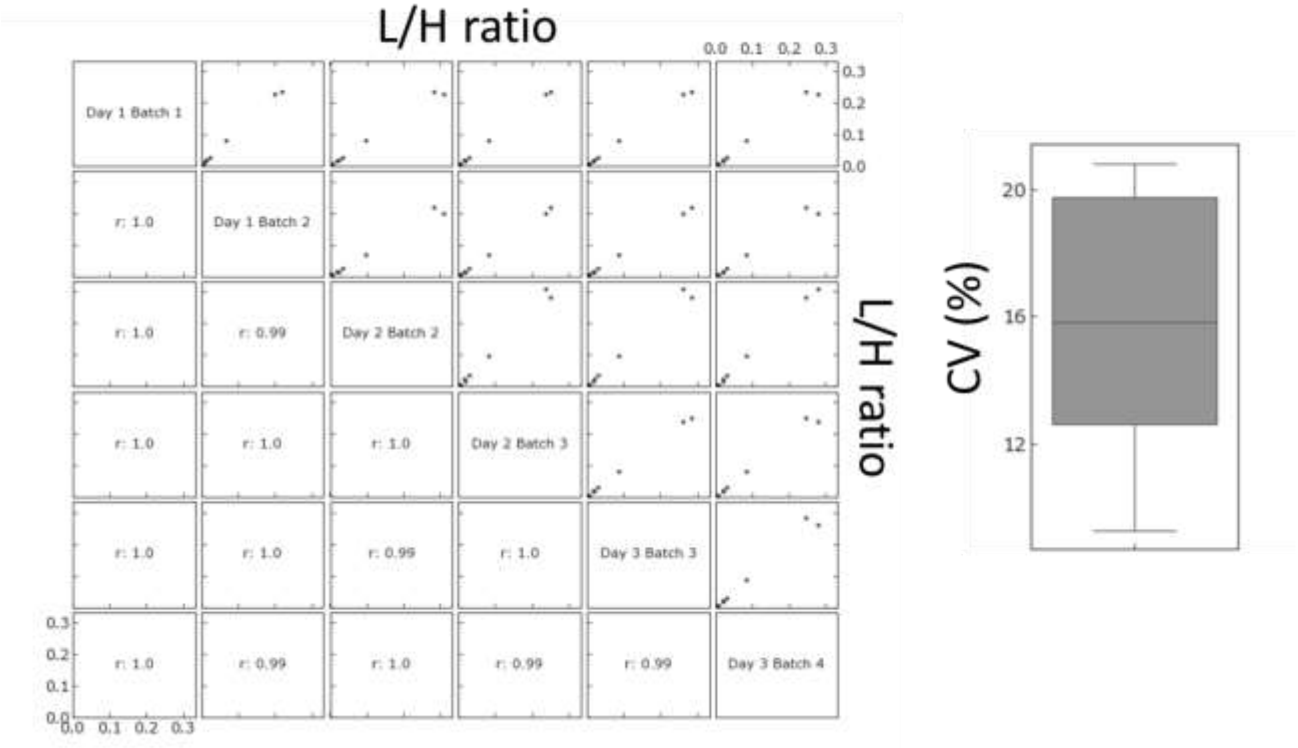

Figure S2. The reproducibility of integrated tip-IMAC for phosphopeptide enrichment. Pearson correlation and CV (%) of peak areas of 10 spiked SIL phosphopeptides (SRM data) in a reference endometrial tumor sample enriched with tip-based IMAC in different days and batches.

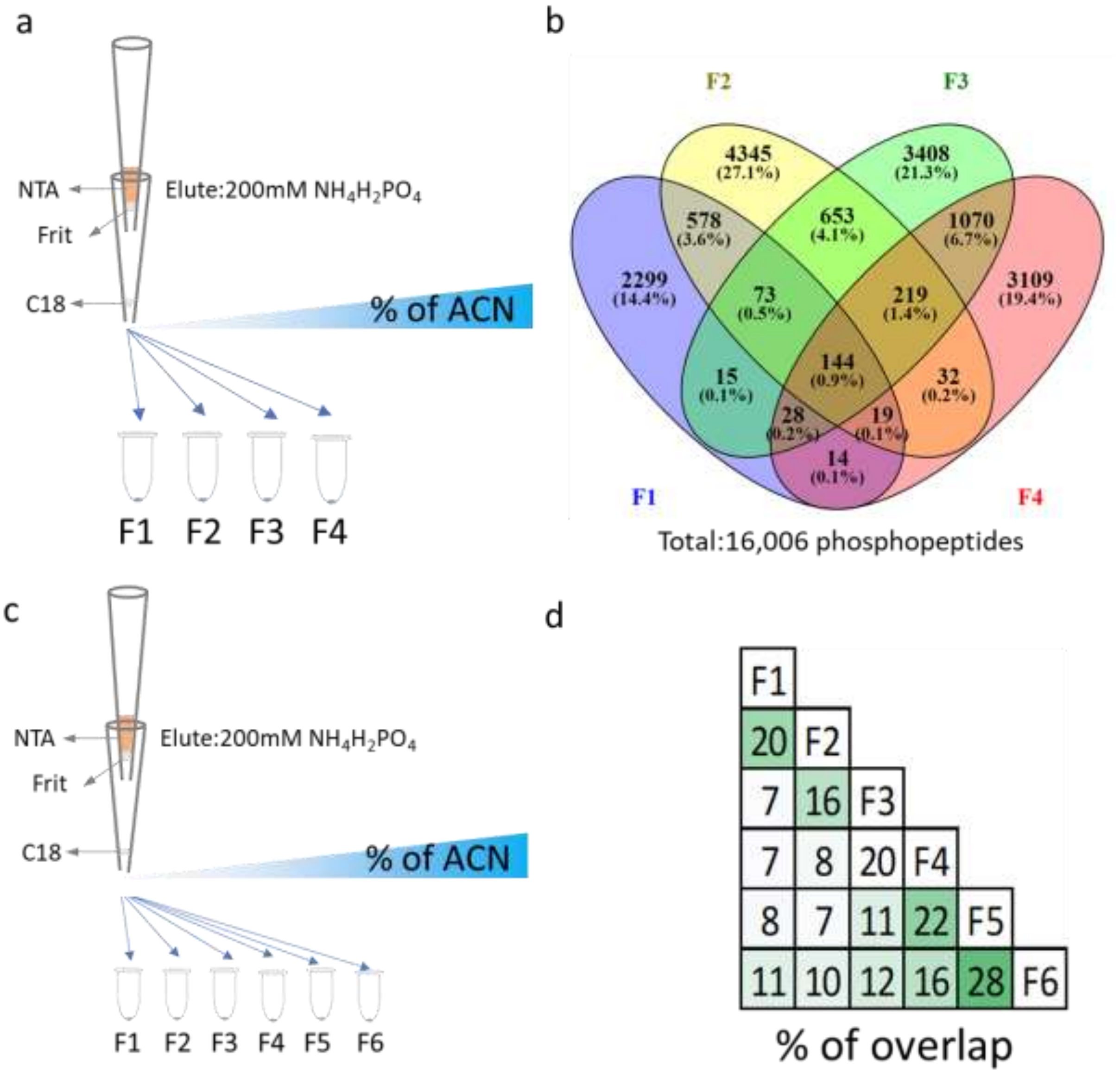

Figure S3. The IMAC-HpH-RP tip method for microscale phosphopeptide fractionation. (a) Using the integrated C18-IMAC-HpH-RP tip method for phosphopeptide fractionation (4 fractions for 20 μg tryptic peptides of MCF-7 cells). (b) Low overlap of the phosphopeptides identified from the 4 different fractions. (c) The tandem IMAC-HpH-C18 tip for phosphopeptide fractionation (6 fractions for 500 μg of tryptic peptides of MCF-7 cells). (d) Separation efficiency calculated by the percentage of common phosphopeptides between fractions.

IT=0.5 s

Pathway	Count	%	PValue
Spliceosome	19	4.52	3.261E-09
RNA transport	15	3.57	8.246E-05
Fc gamma R-mediated phagocytosis	10	2.38	2.087E-04
DNA replication	6	1.43	0.002
Fc epsilon RI signaling pathway	6	1.43	0.026
Proteoglycans in cancer	11	2.62	0.026
mTOR signaling pathway	5	1.19	0.054
AMPK signaling pathway	7	1.67	0.084
Cell cycle	7	1.67	0.086
B cell receptor signaling pathway	5	1.19	0.091
Estrogen signaling pathway	6	1.43	0.098

IT=1.5 s

Pahtway	Count	%	PValue
Spliceosome	33	5.21	2.917E-18
RNA transport	23	3.63	2.324E-07
mTOR signaling pathway	9	1.42	0.001
Cell cycle	12	1.90	0.005
Herpes simplex infection	15	2.37	0.007
Pathogenic Escherichia coli infection	7	1.11	0.010
Proteoglycans in cancer	15	2.37	0.014
mRNA surveillance pathway	9	1.42	0.018
Estrogen signaling pathway	9	1.42	0.028
Ubiquitin mediated proteolysis	11	1.74	0.028
Insulin signaling pathway	11	1.74	0.029
Endocytosis	16	2.53	0.030
MAPK signaling pathway	16	2.53	0.043
Protein processing in endoplasmic reticulum	12	1.90	0.045
Acute myeloid leukemia	6	0.95	0.052
Viral carcinogenesis	13	2.05	0.071
AMPK signaling pathway	9	1.42	0.080
Transcriptional misregulation in cancer	11	1.74	0.084
Fc gamma R-mediated phagocytosis	7	1.11	0.086
Glycolysis / Gluconeogenesis	6	0.95	0.097
Tight junction	7	1.11	0.097
Ribosome biogenesis in eukaryotes	7	1.11	0.097

Figure S4. Pathway enrichment analysis of significantly changed phosphopeptides for 10 ng tryptic digests of AML cells under different ion injection times.

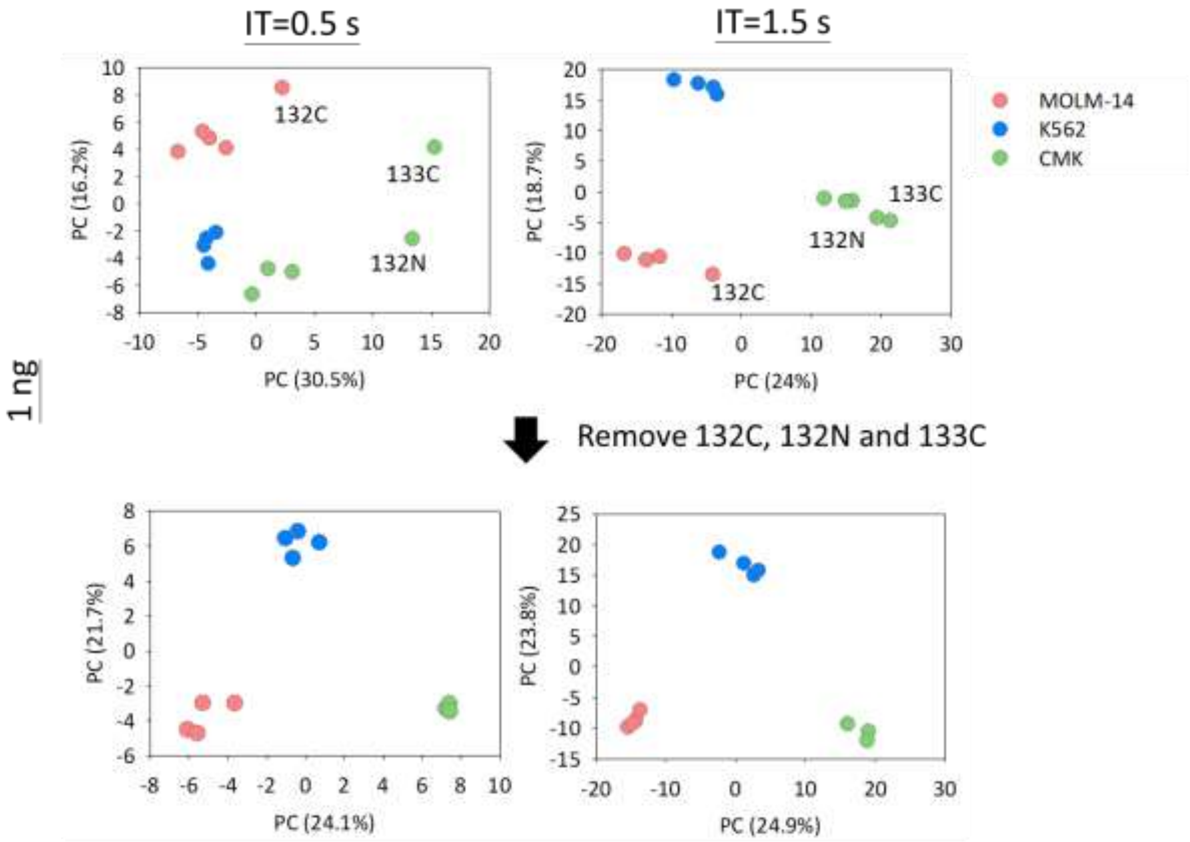

Figure S5. Effect of ion injection time on quantitation quality in the mimic nanoscale phosphoproteome analysis. The PCA analysis of quantified phosphopeptides of 1 ng tryptic digests of the AML cells under two different ion injection times (0.5 and 1.5 s).

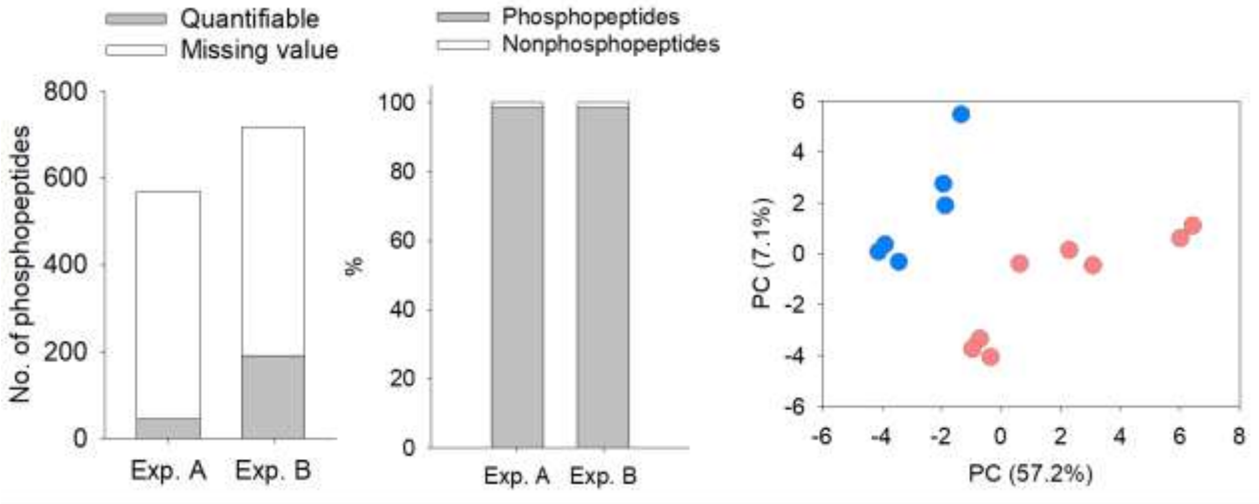

Figure S6. Phosphoproteome analysis for 10 sorted MCF10A cells. (a) The numbers of quantified (70% no-missing value in study samples) phosphopeptides and enrichment specificity in each TMT experiment. (b) PCA analysis shows the clustering of cells from the two different treatment conditions.

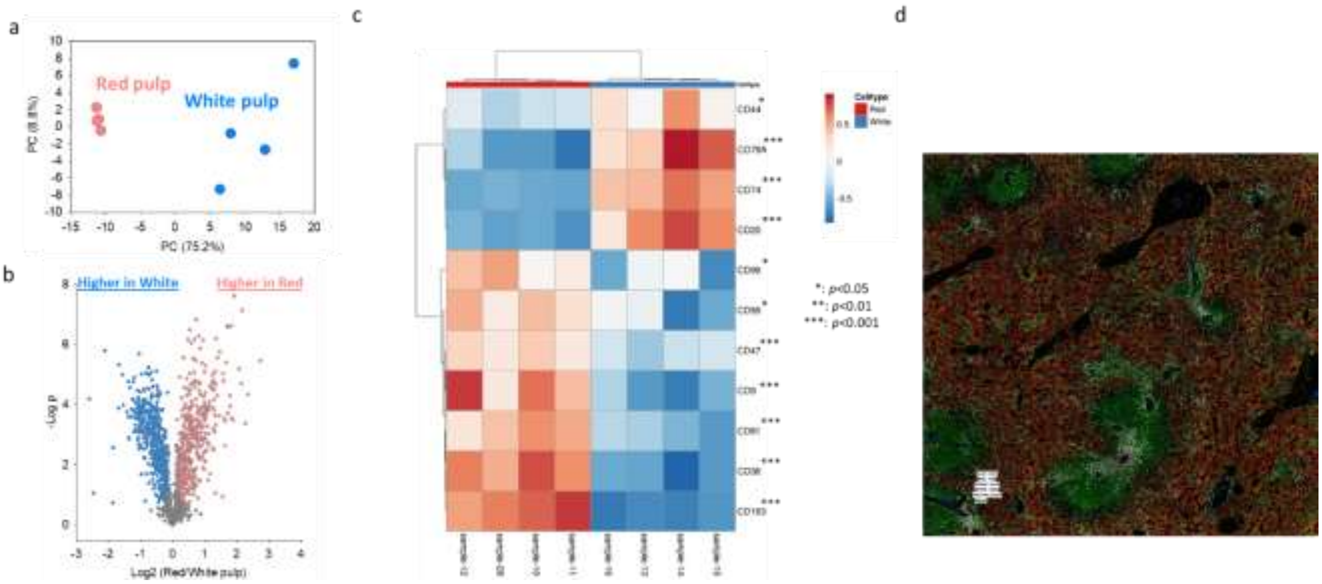

Figure S7. Global proteome analysis of LCM-dissected human spleen tissue voxels. (a) PCA of the proteome data. (b) Volcano plot shows significantly changed proteins in white pulp and red pulp (t-test, n=4 for each condition. s0=1 and FDR=0.05% were used as cut-off values). (c) The altered proteins expression of surface markers. (d) Representative CODEX image of human spleen tissue: CD8α (red), CD163 (yellow), CD3e (white), CD20 (green), CD31 (blue). The image size is 560 μm x 560 μm as shown.

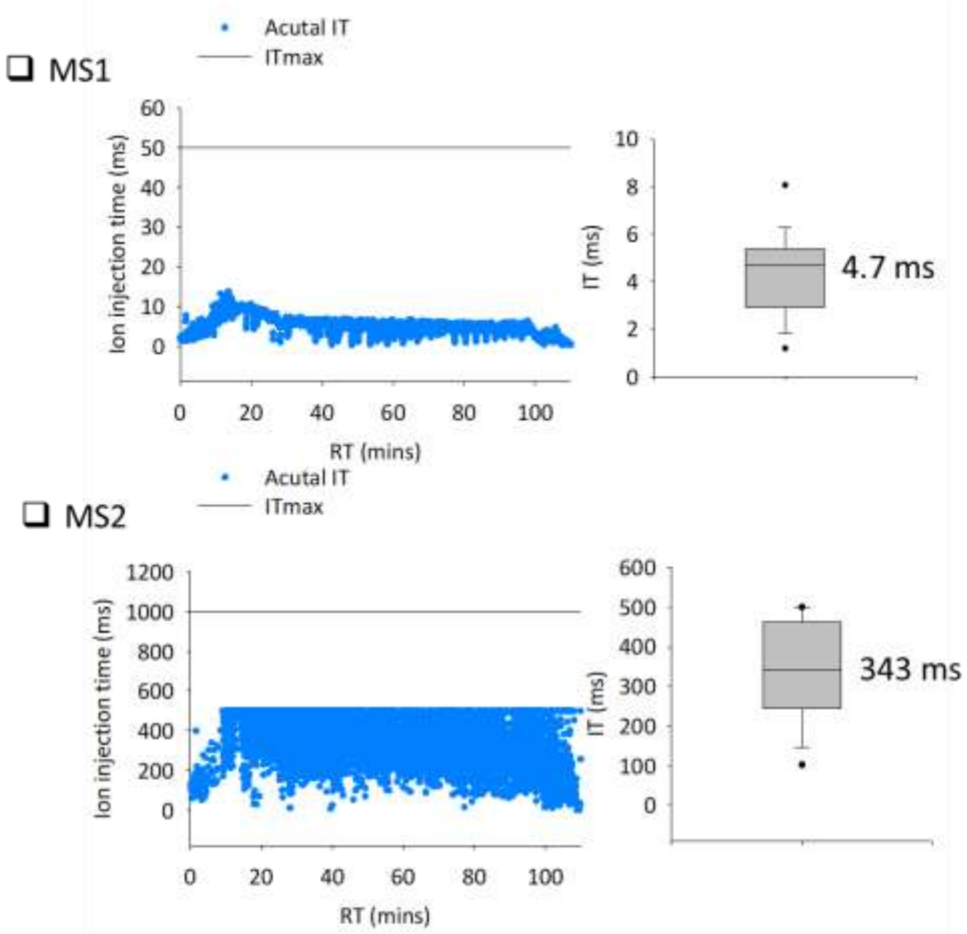

Figure S8. The ion injection time distribution of purified phosphopeptides from 0.1 μg proteins from the A549 cell lysate at MS1 and MS2 level.

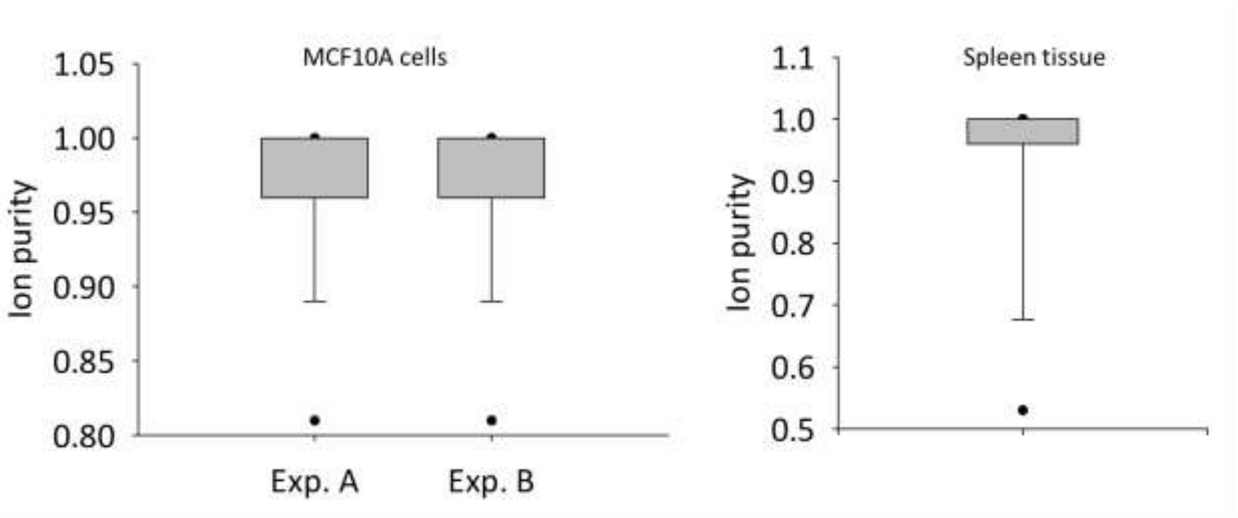

Figure S9. The ion purity distribution of detected phosphopeptides in Figures 6 and 7.
