# Supplementary material for: A streamlined tandem tip-based workflow for sensitive nanoscale phosphoproteomics
Source: Commun Biol. 2023 Jan 18;6:70. doi: 10.1038/s42003-022-04400-x (PMC9849344; doi:10.1038/s42003-022-04400-x)
Supplement: Supplementary file 2 — Description of Additional Supplementary Files [file 42003_2022_4400_MOESM2_ESM.pdf]

## Description of Additional Supplementary Files

**File name:** Supplementary Data 1
**Description:** The raw files and corresponding samples, experimental conditions and MS instrument setting.

**File name:** Supplementary Data 2
**Description:** Pathway enrichment analysis of significantly changed phosphopeptides between EGF- and mock-treated cells. Identification results in Exp. A (a) and B (b). (c) Quantitation results. (d) KEGG pathway. (e) Reactome pathway

**File name:** Supplementary Data 3
**Description:** Pathway enrichment analysis of significantly changed phosphopeptides. (a) Identification results. (b) Quantitation results. (c) Pathways enriched in red pulp. (d) Pathways enriched in white pulp.

**File name:** Supplementary Data 4
**Description:** The summary of the number of identified phosphopeptides in this study and other two published works.

**File name:** Supplementary Data 5
**Description:** Buffer composition for in-Tip High-pH fractionation and concatenation.
