# Supplementary material for: A streamlined tandem tip-based workflow for sensitive nanoscale phosphoproteomics
Source: Commun Biol. 2023 Jan 18;6:70. doi: 10.1038/s42003-022-04400-x (PMC9849344; doi:10.1038/s42003-022-04400-x)
Supplement: Supplementary file 8 — Reporting Summary [file 42003_2022_4400_MOESM8_ESM.pdf]

nature portfolio

Corresponding author(s): Chia-Feng Tsai, Tujin Shi, Tao Liu

Last updated by author(s): Nov 30, 2022

Reporting Summary

Nature Portfolio wishes to improve the reproducibility of the work that we publish. This form provides structure for consistency and transparency in reporting. For further information on Nature Portfolio policies, see our Editorial Policies and the Editorial Policy Checklist.

Statistics

For all statistical analyses, confirm that the following items are present in the figure legend, table legend, main text, or Methods section.

n/a	Confirmed	
☐	☒	The exact sample size (*n*) for each experimental group/condition, given as a discrete number and unit of measurement
☐	☒	A statement on whether measurements were taken from distinct samples or whether the same sample was measured repeatedly
☐	☒	The statistical test(s) used AND whether they are one- or two-sided<br>*Only common tests should be described solely by name; describe more complex techniques in the Methods section.*
☒	☐	A description of all covariates tested
☒	☐	A description of any assumptions or corrections, such as tests of normality and adjustment for multiple comparisons
☐	☒	A full description of the statistical parameters including central tendency (e.g. means) or other basic estimates (e.g. regression coefficient) AND variation (e.g. standard deviation) or associated estimates of uncertainty (e.g. confidence intervals)
☐	☒	For null hypothesis testing, the test statistic (e.g. *F*, *t*, *r*) with confidence intervals, effect sizes, degrees of freedom and *P* value noted<br>*Give P values as exact values whenever suitable.*
☒	☐	For Bayesian analysis, information on the choice of priors and Markov chain Monte Carlo settings
☒	☐	For hierarchical and complex designs, identification of the appropriate level for tests and full reporting of outcomes
☒	☐	Estimates of effect sizes (e.g. Cohen's *d*, Pearson's *r*), indicating how they were calculated

Our web collection on statistics for biologists contains articles on many of the points above.

Software and code

Policy information about availability of computer code

Data collection	Commercial MS software Xcalibur was used to collect all RAW data.
Data analysis	The freely-available open-source Fragpipe software was used for protein identification and quantification. Biological functions and signaling pathways were analyzed by using DAVID Bioinformatics Resources (Version 6.8) and Peruses (Version 1.6.2.1), and protein-protein association network analysis was performed by STRING (Version 11.0).

For manuscripts utilizing custom algorithms or software that are central to the research but not yet described in published literature, software must be made available to editors and reviewers. We strongly encourage code deposition in a community repository (e.g. GitHub). See the Nature Portfolio guidelines for submitting code & software for further information.

Data

Policy information about availability of data

All manuscripts must include a data availability statement. This statement should provide the following information, where applicable:

- Accession codes, unique identifiers, or web links for publicly available datasets
- A description of any restrictions on data availability
- For clinical datasets or third party data, please ensure that the statement adheres to our policy

The RAW MS data and the identified results from Fragpipe have been deposited in Japan ProteOme STandard Repository (jPOST: https://repository.jpostdb.org/)66. The accession codes: JPST001468 for jPOST and PXD032019 for ProteomeXchange. The access link is https://repository.jpostdb.org/

preview/189170729262298fbf5daee and access key is 4830. SRM results including the assay characterization data are organized as Skyline files on the Panorama server67 and can be accessed via https://panoramaweb.org/nanoscale_phosphoproteomics.url. Here are the reviewer account details:Email:panorama +reviewer108@proteinms.net; Password:oHgxvRzH.

Human research participants

Policy information about studies involving human research participants and Sex and Gender in Research.

Reporting on sex and gender	N/A
Population characteristics	N/A
Recruitment	N/A
Ethics oversight	N/A

Note that full information on the approval of the study protocol must also be provided in the manuscript.

Field-specific reporting

Please select the one below that is the best fit for your research. If you are not sure, read the appropriate sections before making your selection.

☒ Life sciences ☐ Behavioural & social sciences ☐ Ecological, evolutionary & environmental sciences

For a reference copy of the document with all sections, see nature.com/documents/nr-reporting-summary-flat.pdf

Life sciences study design

All studies must disclose on these points even when the disclosure is negative.

Sample size	No sample-size calculation was performed. Two types of tissue regions were used for method development to demonstrate broad utility of our method.
Data exclusions	No data were excluded.
Replication	All attempts at replication were successful.
Randomization	This is not relevant to this study.
Blinding	Blinding was not relevant to this study (method development and demonstration).

Reporting for specific materials, systems and methods

We require information from authors about some types of materials, experimental systems and methods used in many studies. Here, indicate whether each material, system or method listed is relevant to your study. If you are not sure if a list item applies to your research, read the appropriate section before selecting a response.

Materials & experimental systems

n/a	Involved in the study	
☒	☐	Antibodies
☒	☐	Eukaryotic cell lines
☒	☐	Palaeontology and archaeology
☒	☐	Animals and other organisms
☒	☐	Clinical data
☒	☐	Dual use research of concern

Methods

n/a	Involved in the study	
☒	☐	ChIP-seq
☒	☐	Flow cytometry
☒	☐	MRI-based neuroimaging
